# Asthma and stroke: a narrative review

**DOI:** 10.1186/s40733-021-00069-x

**Published:** 2021-02-19

**Authors:** A. Corlateanu, Iu Stratan, S. Covantev, V. Botnaru, O. Corlateanu, N. Siafakas

**Affiliations:** 1 Department of Internal Medicine, Division of Pneumology and Allergology, Nicolae Testemitanu State University of Medicine and Pharmacy, Stefan cel Mare street 165, 2004 Chisinau, Republic of Moldova; 2 Department of Internal Medicine, Nicolae Testemitanu State University of Medicine and Pharmacy, Stefan cel Mare street 165, 2004 Chisinau, Republic of Moldova; 3 Department of Thoracic Medicine, University General Hospital, Stavrakia, 71110 Heraklion, Crete, Greece

**Keywords:** Asthma, ACO, Ischemic stroke, Hemorrhagic stroke, SAH, SABA, LABA, ICS

## Abstract

Asthma is a heterogeneous disease, usually characterized by chronic airway inflammation, bronchial reversible obstruction and hyperresponsiveness to direct or indirect stimuli. It is a severe disease causing approximately half a million deaths every year and thus possessing a significant public health burden. Stroke is the second leading cause of death and a major cause of disability worldwide. Asthma and asthma medications may be a risk factors for developing stroke. Nevertheless, since asthma is associated with a variety of comorbidities, such as cardiovascular, metabolic and respiratory, the increased incidence of stroke in asthma patients may be due to a confounding effect. The purpose of this review is to analyze the complex relationship between asthma and stroke.

## Introduction

Asthma is a heterogeneous disease, usually characterized by chronic airway inflammation, bronchial reversible obstruction and hyperresponsiveness to direct or indirect stimuli. It is a problem worldwide with estimated 495,000 deaths every year, thus possessing a significant public health burden [1]. Asthma complications are often the reason for admission to emergency healthcare service and therefore require special attention [2]. Asthma is not curable, but it should be controlled by continuous patient assessment in two domains: symptoms control and future risk of adverse outcomes [1]. Poorly controlled asthma and patients with frequent exacerbations show a greater risk for cardiovascular diseases and ischemic stroke [3, 4]. It is also revealed that the pharmacotherapy of asthma, including β2-agonists and systemic corticosteroids, has implications in the development of asthma comorbidities such as stroke [5, 6]. In addition, as a chronic inflammation, asthma has also a systemic impact by having a correlation with increased atherosclerotic vessel disorders [7]. However, smokers with asthma compared to non-smokers with asthma have frequent asthma symptoms, more medication use, poorer lung function and higher prevalence of comorbidities [3]. This raises the question that stroke in asthmatics may be due to confounding effect (smoking).

Stroke is the second leading cause of death and a major cause of disability worldwide, and there is a further increase in its incidence due to expanding population numbers and aging as well as the increased prevalence of modifiable stroke risk factors [8]. It was demonstrated that stroke may be more frequent in patients with respiratory conditions [9]. Therefore, there may be a significant interplay between asthma and stroke, as it may be an independent risk factor for stroke, and its severity exhibits a linear response of stroke development [10]. These facts represent the base for development of neuropulmonology, which emphasizes the importance of the interconnection between the central nervous and respiratory systems for optimizing the management of patients wherein these pathologies co-exist, especially in the neurocritical care environment [11].

The purpose of this review is to summarize available data on the association between asthma and stroke and to describe their possible pathophysiological links.

## Method

AC, SIu, SC performed the literature review using the terms “asthma”, “stroke”, “subarachnoid hemorrhage”, “smoking”, “SABA”, “LABA”, “SAMA”, “LAMA”, “corticosteroids”, “TPA”, “antiepileptic”, “seizure”, “hypoxia”, “aspirin”, “beta blockers”, “angiotensin converting enzyme”, “comorbidities” along with the MESH terms. The reference list of the articles was carefully reviewed as a potential source of information. The search was based on Medline, Scopus and Google Scholar engines. Selected publications were analyzed and their synthesis was used to write the review and support the hypothesis of the relationship between asthma and stroke.

## Risk factors

### Shared risk factors between asthma and stroke

**Fig. 1:**
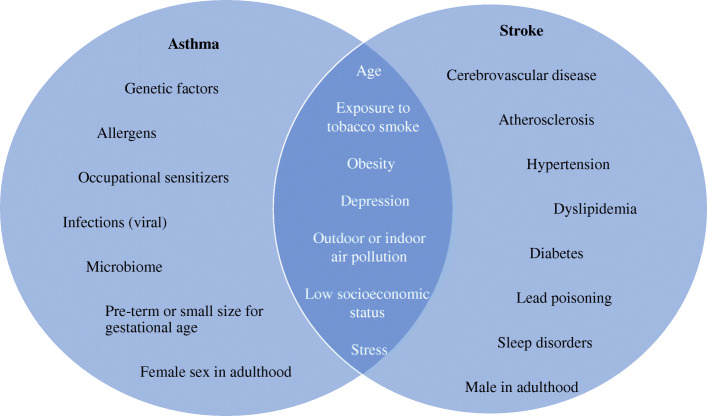
Overlapping risk factors for asthma and stroke

Asthma may be categorized by itself as a risk factor for stroke that is independent of basal lung functioning. It can trigger directly cerebral hypoxemic episodes during asthma attacks or can indirectly increase stroke risk by inducing prothrombotic factors and endothelial dysfunction, thus initiating the development of atherothrombosis [12]. The major risk factors for stroke are history of hypertension, diabetes mellitus, cerebrovascular disease; tobacco exposure, older age, stress, depression, sleep disorders, obesity [13]. Some of these risk factors can also be seen in asthma patients and thus the link between asthma and stroke can be to some degree due to confounding effect (Fig. 1).

A nationwide population-based cohort study was conducted in an Asian population to investigate the effects of asthma on the risk of stroke. The people enrolled in the National Health Insurance program represented the data source, divided into 2 cohorts: patients with newly diagnosed asthma that received treatment (without stroke baseline), were matched for age, sex and index year with 4 reference subjects without asthma. The risk of stroke was analyzed using Cox proportional hazard regression models. The overall incidence of stroke was greater in the asthmatic cohort than in the non-asthmatic cohort (HR = 1.53, 95% CI = 1.47–1.60) with an adjusted HR of 1.37 (95% CI = 1.27–1.48) when adjusting for age, sex and comorbidities [10]. Similar results were registered in 2020 in the HUNT study wherein participants with active asthma showed evidence for a modest increased risk for stroke (adjusted HR 1.17, 95% CI = 0.97–1.41) [3].

Conversely, a recent Korean study did not find increased ischemic stroke risk among asthma subjects (HR = 0.91, 95% CI = 0.86–0.95) [14]. However, there was a significantly higher risk of stroke among asthma patients who encounter more than 3 exacerbations per year (HR = 3.05, 95% CI = 2.75–3.38) [10].

### Stroke subtypes and asthma

A recent meta-analysis on stroke risk in asthma patients that included five articles comprising 524,637 participants and 6031 stroke cases demonstrated that asthma was associated with a significantly increased risk for (see all similar) developing stroke [15]. However, it is not clear whether the increased risk persists for all stroke subtypes. The nationwide study on Asian population revealed that patients with asthma were 1.38 fold more likely to develop ischemic stroke (95% CI = 1.27–1.49) and were 1.31 fold more likely to develop hemorrhagic stroke (95% CI = 1.09–1.65) than were the non-asthmatic controls after adjusting for age, sex, and comorbidities. Thus, incidence of both subtypes of stroke are increased in asthma, especially in those with more than three annual exacerbations [10]. However, the data on subarachnoid hemorrhage (SAH) and asthma is limited mostly presented as case reports [16, 17]. In a prospective cohort study of 20,534 men and 7237 women that lasted 26 years baseline lung function, expressed as low FEV1 or FEV1/FVC, was a risk factor for SAH, independently of smoking [18]. These results suggest that asthma patients may also be at risk for SAH and this depends on degree of obstruction. Therefore, it seems that the current evidence demonstrates an increased risk of all major stroke subtypes in patients with asthma.

### Impact of smoking on stroke risk among asthma patients

One of the main risk factor for death after stroke is smoking [19]. However, the impact of tobacco smoking on health is not limited to those who smoke, but they also affect those in the vicinity who are exposed to secondhand smoke (SHS) [20]. One of the largest number of deaths attributable to SHS in adults is caused by CAD and stroke [21]. Current smoking is linked to poorer outcomes of asthma treatment and thus for more frequent exacerbations and medication use, that represents a major additional risk factor for stroke as will be discussed below. In addition, a cohort study on Copenhagen general population emphasizes the substantial role of tobacco smoking in development of asthma’s cardiovascular comorbidities, through comparison of never smokers asthma patients with former or current smokers asthma patients. Adjusted hazard ratios for ischemic heart disease were 1.2 (0.9–1.6) in never smokers, 1.5 (1.2–2.0) in former smokers, and 2.0 (1.4–2.9) in current smokers. Similar results were found for ischemic stroke 1.4 (0.9–2.1) in never smokers, 1.2 (0.8–1.9) in former smokers, and 3.0 (1.7–5.3) in current smokers [22]. Also, we should mention that smoking is highly associated with COPD, that represent by itself an independent risk factor for stroke [23]. Asthma and COPD can occur concurrently and is termed as asthma-COPD overlap (ACO). Acute exacerbation of ACO may aggravate hypoxemia and inflammation of blood vessels, which are the key risk factors for CHD and stroke [24]. Thus, excess of stroke risk in individuals with asthma and smoking could be partly due to ACO or misclassification of COPD as asthma in smokers [3]. These results bring into light the importance of smoking cessation as a first line action in asthma treatment, since the prevalence of tobacco smoking is similar in individuals with asthma, as it is in the general population [25].

## Pathogenesis and pathophysiology

### Atherosclerosis is the main pathophysiological mechanism of stroke development in asthma?

Asthma has a systemic impact that is associated with the development of atherosclerosis and several studies revealed measurable modifications in the structure and function of blood vessels. In details, central pulse wave velocity has the highest values in severe asthma cohort (*p* = 0.005); vascular strains presented a relevant decrease of circumferential and radial strains in severe asthma (3.18 ± 0.23%, 3.47 ± 0.20%, respectively) in comparison to controls (4.29 ± 0.35%; *p* = 0.013) [7]. Brachial-ankle pulse wave velocity measurement is a marker of early atherosclerotic changes that was assessed in a cohort study, demonstrating an increase in baPWV in patients with asthma compared with control subjects [26]. Also, asthma was not only associated with preatherosclerotic vessel alterations, such as higher arterial stiffness, but much more with increased prevalence of manifested atherosclerosis compared to non-asthma individuals. Specifically, atherosclerotic plaques were seen in 43.1% of patients with severe asthma, 25% of mild-to-moderate asthma and 14.3% of control study participants (*p* = 0.035) [7]. Subclinical atherosclerosis in asthmatic patients was described in a cross-sectional study, through measurement of carotid and femoral intima thickness, which both were significantly higher in patients with asthma compared to control groups (5.52 ± 0.4 mm vs 5.36 ± 0.4 mm; *p* = 0.038 and 5.64 ± 0.4 mm vs 5.46 ± 0.5 mm; *p* = 0.036, respectively) [27].

Underlying mechanisms in initiating atherosclerosis seems to be related to the hypercoagulable state of asthma. Bazan-Socha and coworkers demonstrated the increase in both thrombin generation and platelet activation and the decrease in fibrinolysis. Asthma patients had 20.0% increased endogenous thrombin potential and 14.4% longer clot lysis time (*p* = 0.001) associated with 21.3% higher plasminogen activator inhibitor-1 [28]. Furthermore, they investigated whether this prothrombotic state is due to chronic inflammation, and showed that asthma was characterized by 62% higher plasma Il-6 and 35% higher TNFα, along with higher CRP, fibrinogen, as well as α2-macroglobulin and PF-4 [29]. Similar results were obtained also by Sneeboer and coworkers, who revealed high levels of PAI-1, D-dimer, von Willebrand factor and plasmin-α2-antiplasmin complexes in asthma [30]. While pro-inflammatory cytokine Il-6 is an inducer of acute phase proteins, such as CRP, hepcidin, fibrinogen, which are the cause of increased thrombin generation, PAI-1 and TNFα are the main regulators of fibrinolysis. PF-4 has implications in platelet activation cascade and α2-macroglobulin seems to counteract the enhanced thrombin dynamics, but can also promote coagulation by binding to protein C and accelerate the cascade [28]. In addition, it was associated an increase in both baPWV and CRP in asthma patients compared with control subjects [26]. There are evidences that CRP is associated mostly with plaque instability [31]. It was found a link between the activity of lipoprotein-associated phospholipase A2 and increased risk of atherosclerosis in asthma patients [32]. The elevated risk of thromboembolic and cardiovascular events in asthma could be also linked to fibronectin, a marker of vascular injury, which is suggested to be a newly determined modulator of prothrombotic plasma properties, and also a sign of the degree of severity of asthma [33].

All the above findings highlights the importance of evaluating the hypercoagulable state of asthmatic patient in order to monitor as predictors of atherosclerotic and thromboembolic events, events that per se can lead to stroke.

### Relationship between FEV1 and stroke risk

The relationship between pulmonary function expressed by forced expiratory volume in 1 s (FEV1) and asthma comorbidities such as CVD or stroke was established by several cohort studies. FEV1 at rest and after response to bronchodilation are the generally accepted surrogate marker of asthma severity. Gulsvik and coworkers conducted an extensive study on 5617 participants, and observed an association between baseline FEV1 and risk of fatal stroke HR = 1.38 (95% CI = 1.11–1.71) and HR = 1.62 (95% CI = 1.22–2.15) for men and women, respectively (adjusted for age and height). The findings could not be explained by smoking, hypertension, diabetes, atherosclerosis, socioeconomic status, obstructive lung disease, physical inactivity, cholesterol or body mass index and persisted in s never-smokers, subgroups without respiratory symptoms and survivors of the first 20 years of follow-up [34]. Similarly, the Atherosclerosis Risk in Communities study which followed 13,842 middle-aged adults initially free of stroke and CHD for 13 years it was demonstrated that white subjects with impaired lung function have a modestly higher risk of ischemic stroke even if they have never smoked nor had respiratory symptoms [35].

However, another cohort study suggested a correlation between FEV1 and arterial stiffness in asthmatic patients. In detail, FEV1 in asthmatics was positively correlated with small arteries elasticity index and negatively correlated with the systemic vascular resistance in these patients. These correlations were not observed in non-asthmatic controls [36]. Moreover, there was a negative correlation between baPWV and FEV1, after adjusting age, gender, BMI and smoking status [26] and both CIMT and FIMT were negatively correlated with FEV1 (r = − 0.417, *p* < 0.001 and r = − 0.294, *p* = 0.007, respectively) [27]. These findings suggest once more the importance of prospective monitoring and treatment of asthma patients.

### Impaired lung function and CVD risk and stroke

The chronic inflammation present in asthma mediates the initiation and progression of atherosclerosis and is intricately involved in plaque rupture and acute CVD events. A large contemporary, multiethnic, long-term, prospective cohort study was conducted by Tattersall and coworkers to analyze the association of asthma and CVD. They found that persistent asthmatics had greater risk of CVD events than non-asthmatics (HR = 1.6, 95% CI = 1.01–2.5), even after adjustment for age, sex, race, CVD risk factors, and antihypertensive and lipid medication use [37]. Furthermore, a cohort study comprising 446,346 Taiwanese adults, showed similar results: an increase of 27% risk of dying from CVD in individuals with active asthma (adjusted HR = 1.32, 95% CI = 1.08–1.62). Additionally, they established that the risk of death from CHD or stroke was increased in a similar manner (HR = 1.16, 95% CI = 0.77–1.73 and HR = 1.23, 95% CI = 0.86–1.74, respectively). Moreover, deaths from CVD and stroke, were stronger associated with active asthma in men than in women [38]. Unlikely, a recent study in Korean adults confirmed a significantly higher prevalence of ischemic heart disease (OR = 1.46, 95% CI = 1.25–1.71) in those with asthma, especially, in older patients and// or untreated asthma patients, but stroke was not significantly associated with asthma (OR = 1.17, 95% CI = 0.92–1.48) in adjusted model [39]. A previous HUNT study concluded that asthma and lack of asthma control were associated with moderately increased risks of atrial fibrillation [40].

Heart diseases, such as acute myocardial infarction, atrial fibrillation and other can induce stroke. These abnormalities are frequent comorbidities in asthma patients. The prothrombotic state encountered in asthma p that we covered above, along with cardiotoxic effects of beta-2 agonists, that we will cover later, are some of the plausible mechanism of CVD due to asthma. Furthermore, we should mention that long-term effects of airway remodeling due to inflammatory response and the subsequent repair mechanism in asthma can induce irreversible airway obstruction and contribute to decreased lung function over time, leading to chronic hypoxia and oxidative stress that may lead to ischemic heart disease [39]. Dysfunction of the airway autonomic nervous system in asthma patients could be an inducer of dysfunctional atrial electrophysiology, causing atrial arrhythmias [41]. Also asthma may be associated with CVD due to other factors such as obesity, smoking, or physical inactivity [39].

Therefore, CVD through multiple factors are a direct risk factor for stroke. Increased tendency for thrombosis may contribute to thrombus formations inside or outside the ventricular cavity, with subsequent cerebral embolization or rupture of a vulnerable plaque in the remote cerebral circulation. Also, high plasma levels of brain natriuretic peptide and D-dimer are independent risk factors for cardioembolic stroke [12]. We, therefore, once again underline the importance of monitoring these parameters in asthma patients.

## Asthma phenotypes, severity and comorbidities

### Asthma phenotypes and stroke

Early-onset asthma and late-onset asthma are 2 substantially different disease phenotypes and differ in risk factors, pathophysiology, answer to treatment, and incidence of comorbidities, such as CVD, stroke. It has been shown by certain cohort studies that late-onset asthmatics had a higher adjusted risk of CVD than non-asthmatics (HR = 1.57, 95% CI = 1.01–2.45) [37]. Furthermore, adult asthma was associated with a 1.40-fold (95% CI = 1.35–1.45) increased hazard of CHD, a 1.20-fold (95% CI = 1.15–1.25) hazard of cerebrovascular disease [42].

In addition, Onufrak et al. conducted a study in which was assessed the correlation between intima-media thickness and adult-onset asthma. They ascertained that the mean CIMT difference between women with adult onset asthma and no history of asthma was attenuated but remained significant (0.713 mm vs. 0.687 mm, *p* = 0.008), thus demonstrating that adult-onset asthma but not child-onset asthma is associated with increased carotid atherosclerosis among women but not among men. Important to mention that, both men and women with history of adult-onset asthma were older, had less education, lower FEV1, more pack/years of smoking, and were more likely to have diabetes and hypertension than their non-asthmatic counterparts. The mechanism of predisposition of women to atherosclerosis is thought to be linked with hormonal effect on leukotriene production. Women with adult onset asthma also had elevated BMI and reported lower leisure physical activity [43], again emphasizing the importance of smoking, obesity, sedentariness as the main risk factors for both asthma and atherosclerosis associated with CVD events and stroke.

It was demonstrated that there is a differential enrichment of genes between adult-onset asthma and childhood-onset asthma. In details, patients with the adult-onset form have more gene signatures associated with eosinophilic airway inflammation, mast cells, and group 3 innate lymphoid cells [44]. It seems that eosinophil cationic protein is a biomarker of coronary atherosclerosis [31]. It could be implemented especially in adult-onset non-atopic, inflammation-predominant asthma phenotype to quantify ECP in order to improve the monitoring of cardiovascular risk.

Adult-onset asthma is characterized by worse prognosis and poorer response to standard asthma treatment, that the cause of elevated use of beta-adrenergic and glucocorticoid drugs [45], that could be a secondary risk for CVD events or worsening of comorbidities in asthma patients, as we will point out below.

### Asthma control, severity and stroke risk

According to GINA report, asthma symptom control represents an important predictor of asthma outcomes. Thus, uncontrolled asthma is the ultimate step to severe asthma and an important risk factor for exacerbations [1]. As it was stated in the HUNT study, patients with not controlled asthma had an increased risk of stroke (HR = 1.34, 95% CI = 1.03–1.73) compared to controlled asthma (HR = 1.34, 95% CI = 1.03–1.73) [3]. Moreover, severe asthma was related to a statistically significant difference in CIMT and FIMT (*p* = 0.002 and *p* < 0.001, respectively) [27] and the highest risk for AF (adjusted HR = 1.74, 95% CI = 1.26–2.42) [40]. Likewise, patients with severe asthma had increased baPWV and CRP compared to patients with stable asthma and control subjects [26].

As we mentioned, there is an obvious risk of atherosclerosis, CVD events in those with severe and uncontrolled asthma. The underlying causes could correlate to the high frequency of comorbid conditions associated with asthma control and severity, [46] more flare-ups, additional medication use with increasing side-effects, and the remodeling process linked to decreased lung function.

Therefore, it is imperative to have a good control on asthma symptoms by a comprehensive therapy.

### Asthma exacerbations and stroke risk

Asthma exacerbations are highly related to further complications, especially myocardial infarction and stroke. Raita and coworkers identified 4607 adults hospitalized for asthma exacerbation who had a first episode of acute myocardial infarction or ischemic stroke. During the reference period, the incidence rate of CVD events was 25.0/100 person-years. In the subsequent risk period of one to 7 days after asthma exacerbations, the incidence rate significantly increased to 129.1/100 person-years with a corresponding adjusted incidence rate ratio of 5.04 (95% CI = 4.29–5.88) [4]. As well, compared with the non-asthmatic cohort, the patients in the asthmatic cohort that visited the emergency room more than 3 times per year were associated with a significantly higher risk of stroke (adjusted HR = 3.05, 95% CI = 2.75–3.38) [10]. Moreover patients who have wheeze attacks with shortness of breath have a greater risk for stroke [47].

There are several potential mechanisms linking asthma exacerbation to the increased incidence of CVD events, including stroke. Specifically, in acute inflammation, thin-cap atheroma could rupture and release inflammatory cells, causing acute accumulation of platelets, neutrophils, and fibrin as well as trapping of red blood cells. Moreover, patients with asthma exacerbation experience hypoxemia, resulting in oxidative stress and insufficient oxygen supply to the myocardium or brain tissue. Also, dysfunction of autonomic nervous system – common mechanism in asthma and coronary vasospasm – could be the cause of myocardial infarction. Furthermore, management of asthma exacerbation, especially excessive use of β2-agonists, necessity of oral or systemic corticosteroids, may have attributed to the subsequent cardiovascular event risks [4].

Among laboratory parameters describing prothrombotic plasma properties, asthmatics with at least one exacerbation were characterized by longer clot lysis time and lower levels of α2-macroglobulin. Both these laboratory variables were also shown as independent predictors of asthma exacerbation in a multiple logistic regression model [48]. As we mentioned previously the counteractive function of α2-macroglobulin, lower levels of these universal protease inhibitor in patients with asthma exacerbations, loose the contributing effect to the attenuation of the prothrombotic state, thus the patients become prone to thromboembolic events.

These findings provide opportunities for clinicians to apply cardiovascular prevention measures (antithrombotics) for patients with severe asthma exacerbation during hospitalization and transition to outpatient care [4].

### ACO and stroke

Chronic obstructive pulmonary disease and asthma are the most frequent chronic respiratory diseases that affect the general population and may sometimes coexist [49]. It is presumed that patients with ACO experience more frequent exacerbations, poorer quality o life, more progressive lung function deterioration, and elevated health care utilization in comparison to asthma or COPD alone. These characteristics, as we explored earlier, dramatically augment the risk of CVD events and stroke. In a cohort study, asthma, COPD and ACO patients were analyzed and differentiated under certain criteria: comorbid conditions, including diabetes, CHD, stroke, were significantly more common in ACO group compared to asthma and COPD groups; the ACO, vs age-matched asthma subgroup had lower prebronchodilator FEV1 (82.1% vs 88%. *P* = 0.017); also ACO group had significantly more asthma attacks in the past year that the age-matched asthma subgroup (49.8% vs 38.4%) and more participants with blood eosinophil counts ≥400 cells/μL (16.9%) vs COPD (9.5%) and asthma subgroup (6.7%) [50].

Yeh et al. evaluated the relation between ACO, neurodegenerative diseases and stroke. They showed that for ACO cohort the incidence rate of stroke (18.5 vs 15.1 per 1000 person-years) were higher than did with the non ACO cohort, with a crude HR of 1.23 (95% CI = 1.15–1.32) The main mechanisms of increased stroke risk are: the high frequency of exacerbations that aggravate systemic inflammation, hypoxemia that trigger oxidative stress - which is presumed to be the main mechanism of neurodegeneration, and which in turn aggravate the existing atherosclerosis [51].

### Asthma comorbidities and stroke

Chronic respiratory diseases are associated with a number of comorbidities due to their proinflammatory state [52]. Asthma is not an exception and there list of commonly encountered comorbidities includes chronic rhinitis, chronic sinusitis/rhinosinusitis, gastroesophageal reflux disease, obstructive sleep apnea/sleep-disordered breathing, psychological disturbances (particularly depression and anxiety disorders), chronic/recurrent respiratory infections, hyperventilation syndrome, hormonal disturbances and other [53]. There are also possible emerging comorbid conditions such as cardiovascular, obesity, metabolic syndrome, diabetes mellitus, degenerative joint disease/arthritis and psychiatric diseases [53, 54]. Some of these comorbidities lead to an increased risk of stroke and are highly prevalent in asthma patient (Table 1). This raises the question that the increased risk of stroke in asthma patients may be due to confounding effect. Nevertheless, the important point is that proper screening and diagnosis of comorbidities in asthmatics is essential for preventing serious complications including stroke.

**Table 1:** Prevalence of comorbidities in asthmatics and risk of developing stroke

Comorbidity	Prevalence in asthma patients	Risk of stroke in non-asthmatics
Hypertension	12–40% [55]	in treated controlled group aHR was 2.21 (95% CI, 1.01–4.82), in untreated hypertension group 2.55 (95% CI, 1.93–3.37), in treated uncontrolled group 4.30 (95% CI, 3.16–5.85) [56]
CAD	7.2–12.9% [57, 58]	HR of 1.8 (95% CI, 1.03–3.43) [59]
Atrial fibrillation	3.8–8.95% [60, 61]	aHR 3.13 (95% CI, 1.50–6.56) [62]
Obesity	21–48% [63–66]	OR 1.57, (95% CI,1.28–1.94) [67]
Diabetes mellitus	8.4%-31.1 [68, 69]	aHR 1.75, 95% CI, 1.64–1.86) [67]
OSAS	40–50% [70, 71]	HR 2.52, (95% CI, 1.04–6.01) [72]
GERD	25.4–82% [73, 74]	1.68-times more likely (95% CI, 1.03–2.76) [75]

## Medication

### Impact of asthma treatment on stroke

Asthma treatment, including bronchodilators and oral or systemic corticosteroids, has been identified as risk factor for CVD events and stroke, whereas, inhaled corticosteroids showed a protective effect. Compared with asthmatic patients who received inhaled corticosteroids the patients who received inhaled SABA or LABA had a significantly increased risk of stroke (aHR-193, 95% CI = 163–227), followed by those who had received both inhaled corticosteroids and inhaled SABA or LABA treatment (aHR = 133, CI = 113–156) [10]. Also, carotid atherosclerosis is reduced in asthmatic patients treated with ICS compared with matched controls, this study suggests that ICS may have protective effects against atherosclerosis [76].

Bronchodilators are important in the management of asthma because they play an essential role in reversing airway obstruction and provide “bronchoprotection” against bronchospasm due to exercise and other spasmogenic stimuli, although the current view is that asthma treatment with a bronchodilator should never be started in the absence of an ICS [1]. Contrary ICS, use of oral corticosteroids, alone or in combination, was associated with greatly enhanced risk of CHD (HR = 2.59, 95% CI = 2.49–2.69), cerebrovascular disease (HR = 1.91, 95% CI = 1.81–2.01) and heart failure (HR = 3.48, 95% CI = 3.34–3.63) [42]. Oral and systemic corticosteroid therapy comes with known risks for acute and chronic complications, including hypertension, metabolic syndrome, osteoporosis, weight gain, cataracts, gastrointestinal bleeds, impaired wound healing and psychological disorders. Adults receiving SCS treatment had greater odds of complications and greater associated costs over 3 years than matched non-SCS asthma patients [5]. The adjusted OR for myocardial infarction in current users of oral corticosteroids compared to non-users was 1.42 (95% CI = 1.17–1.72) [77]. Asthmatic patients have a prothrombotic state that increases with asthma severity. This prothrombotic state is most likely caused by chronic airway inflammation as we pointed above, and combined with the effect of high-dose corticosteroids and might explain the increased risk of patients with severe asthma to have venous thromboembolism [30]. Most patients with severe asthma are exposed to SCS, which increase SCS-related adverse effects risk. This suggests that SCS exposure should be minimized as recommended by asthma treatment guidelines [78].

Recent increases in understandings of the mechanisms of asthma and new biomarkers have led to development of potentially more targeted therapy for the management of severe asthma, supplanting the use of long-term steroids and thereby bypassing steroid-related adverse events [79]. Benralizumab administration for 28 weeks significantly reduced oral glucocorticoid dose by 75% compared with placebo, with about half of subjects receiving baseline prednisone doses of less than or equal to 12.5 mg/d stopping steroids completely [80]. Dupilumab reduced the rate of severe exacerbations, improved lung function, and improved quality of life in patients with uncontrolled persistent asthma receiving medium- to high-dose ICSs and LABAs [81]. Furthermore, FEV1 increased after 52 weeks in the low-, medium-, and high-dose tezepelumab groups compared with the placebo group [82]. Nowadays, pharmacological research has promised safe and effective therapeutic options for patients with severe, uncontrolled asthma, a very complex and heterogeneous entity [83].

However, a follow-up analysis evaluated the risk of serious cardiovascular and cerebrovascular adverse events and showed a higher crude incidence of these events in omalizumab-treated patients (13.4 per 1000 person-years) compared with non-omalizumab-treated patients (8.1 per 1000 person-years) [84].

Specific potential adverse cardiovascular effects are more frequent with β2-agonists, including circulatory disturbances via hypokalemia, prolongation of depolarization-repolarization (QT) interval and sinus tachycardia and interference with cardiovascular autonomic control [85]. There is an apparent association between altered autonomic cardiovascular control and asthma. This relationship is twofold: a consequence of both the pathophysiology of asthma per se and the effects of asthma pharmacotherapy. Consequently, it is possible that these drugs might be implicated in the pathogenesis of a number of CVD risk factors, including insulin-resistance, hypertension and cardiovascular hypertrophy and in the evolution of CHD, cerebrovascular disease and sudden death [41]. In detail, a nationwide population-based nested case-control study in Taiwan documented that inhaled bronchodilators were independently associated with an increased risk of atrial fibrillation. New users of bronchodilator had the highest risk of atrial fibrillation during first 6 m [86]. Actually, β2-agonists have been described as a direct or indirect potential mechanism of death in asthmatics; they can induce increased risk of myocardial infarction, congestive heart failure, cardiac arrhythmia/arrest and sudden cardiac death with particularly high-risk of cardiac event in patients with long-QT syndrome [87]. However, an Australian population based cohort study reported significant associations of incident cardiovascular disease and stroke events in both male and female subjects with traditional risk factors, including use of a SABA. These events were positively associated with as-needed SABA use (OR, 2.66) but not at least once-daily use (OR, 0.81), and there was an inverse and non-significant association of LABA use alone or in combination with ICSs (OR, 0.58), although incident events were correlated with asthma and LABA use with or without an ICS in female subjects [88]. Furthermore, a nested case-control study showed that the use of inhaled ipratropium was associated with an increased risk of arrhythmia in adolescents and young adults with asthma compared to non-users, although the absolute risk was low [89]. Theophylline can cause tachycardia and serious arrhythmias even at serum theophylline concentrations considered to be therapeutic. Multifocal atrial tachycardia, an arrhythmia associated with use of this drug, may herald sudden cardiac death. However, there is evidence that doxofylline could offer a promising alternative to theophylline with a superior efficacy and safety profile in the management of patients with asthma [90]. In young, otherwise healthy asthmatic subjects, combined therapy with theophylline and an oral β-adrenergic agonist (terbutaline) does not lead to an increase in the total number of ectopic beats but may increase the degree of complexity of ventricular premature beats [91]. This effect could be partly due to hypokalemia and hypomagnesemia were more prevalent among asthmatics that received β2-agonist in either monotherapy or combined with steroid and or theophylline [92].

Nonetheless, both β-agonists and xanthines have direct effects on the human lower esophagus. These actions most probably are due to an inhibitory effect on active resting tension in the circular muscle layer of the human esophago-gastric junction. Actually, inhaled salbutamol reduces lower esophageal sphincter basal tone and contractile amplitudes in the smooth muscle esophageal body in a dose-dependent manner and may increase the likelihood of acid reflux at least in patients who receive cumulative dosing, and theophylline treatment causes a significant increase in total reflux time and reflux symptoms but does not worsen asthma [93]. Anticholinergic agents not only influence lower esophageal sphincter performance, but also affect other activities in the gastrointestinal tract that are involved in the etiology of reflux. They decrease saliva secretion, esophageal peristalsis, TLOSRs, gastric emptying, and gastric acid production [94].

Alternatively, Lee and coworkers showed that inhaled respiratory treatments including LAMA have no effects on the development of stroke. LAMA use was not significantly associated with any increase in the risk of stroke in total study group (in total LAMA; aOR, 0.97; 95% CI, 0.90–1.05) or any subgroup. After adjusting for covariates, there were no statistically significant effects of inhaled drugs on the stroke incidence. All of the aOR ranges were between 0.97 and 1.08. The inhaled bronchodilators did not affect either hemorrhagic or ischemic strokes. However, ICS without LABA was statistically significantly associated with hemorrhagic stroke (aOR, 1.51; 95% CI, 1.01–2.25) [95].

### Impact of stroke treatment on asthma

Several medications are used in treatment and prophylaxis of stroke. Although, it is not commonly underlined but some of them can have positive and negative effects in asthma patients.

Tissue plasminogen activator (TPA) is the main drug that revolutionized the management of ischemic stroke. As with any other drug there have been cases of anaphylactic reaction which require emergency treatment. Allergic reactions included angioedema, facial swelling, urticaria, skin rash, cutaneous hypesthesia, hypotension, anaphylactic shock, and death [96]. The true incidence of these events is hard to assess. Out of 924 adverse events only 12 cases were directly attributed to IV thrombolytic medication. Eleven cases were due to IV alteplase and one due to IV reteplase [96]. Asthma patients are generally more likely to have allergic events due to the atopic nature of their disease [97, 98]. TPA is an essential lifesaving medication for ischemic stroke that is associated with an improvement of quality of life and general prognosis of the disease [99, 100]. However, as asthma patients are prone to allergic events general awareness of the possible side effects are important to consider in selective cases. Mild allergic reaction that involve skin and subcutaneous tissue generally responds well to steroids and antihistamine drugs, however they should not be confused with acute anaphylaxis which requires epinephrine [101, 102]. In cases of orolingual angioedema the general steps in the management are stopping the TPA infusion, diphenhydramine, ranitidine/famotidine, methylprednisolone, epinephrine, and otolaryngology or anesthesia consult with the assessment of airways every 15–20 min [103–105].

Aspirin and non-steroidal anti-inflammatory medication are well known drugs that can trigger allergies particularly in asthma patients (Samter’s triade) [106]. This limits the use of aspirin as an antithrombotic drug in clinical practice as a method to achieve optimum medical management prior to and after neurointerventional treatment [107]. In this group of patients aspirin desensitization therapy can be used to overcome this problem and have proven their effectiveness in cardiovascular and cerebrovascular disease [107, 108]. Clopidrogel and ticlopidine allergies can be managed in a similar fashion [109].

β-blockers are frequently used to manage arrhythmias, hypertension and other cardiovascular disease. Traditionally, they are contraindicated in patients with asthma as they may lead to bronchoconstriction and exacerbate the condition. However, there is more data on the use of β-blockers in fundamental and clinical practice [110]. There is a debate between the use of selective versus non-selective β-blockers as some studies indicate that selective β-blockers possess less risk for asthma patients [111, 112]. Therefore, it seems that β-blockers can be used more widely when they are indicated but when the cardiovascular risks overweight the risk for pulmonary complications [113]. Another group of drugs frequently used to manage hypertension are angiotensin converting enzyme inhibitors. Although, that they do not cause changes of pulmonary function they can cause cough and wheezing which can be interpreted as asthma manifestation [114]. Therefore angiotensin-receptor blockers are a better alternative for asthma patients [115]. Hypertension can also be managed with calcium channel blockers. They are not contraindicated in asthma and in some types of asthma can event be beneficial for lung function improvement [116].

Seizures are a frequent complication in stroke patients. Interestingly, asthma patients have a higher risk of epilepsy [117]. This makes them particularly at risk of stroke-associated seizures. Some antiepileptic drugs might play roles in preventing or reducing the frequency of asthma attacks particularly phenytoin, valproic acid and carbamazepin [118–120]. Interestingly, lidocaine that works primarily by blocking sodium channels and decreasing membrane excitability is effective in a form of nebulizer for treating asthma patients [121]. Although, this group of medications is not the standard of care in asthma, reports of antiepileptic drug efficiency raises several important questions that some patients have a neurogenic component to their disease, particularly in a form of channelopathies.

## Disease progression, relationship and prevention

### Overall pathophysiological mechanisms from asthma to stroke

**Fig. 2:**
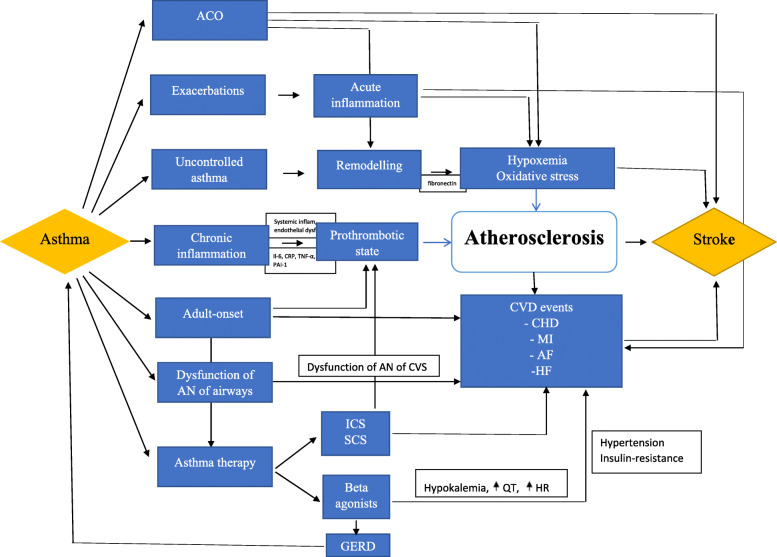
Atherosclerosis as a major pathophysiological mechanism in development of stroke in asthma patients

Based on the evidences presented above we may argue that atherosclerosis could be the main pathophysiological mechanism in development of stroke in asthma patients, including the facts analyzed previously (Fig. 2). This concept is also sustained by other studies which try to explain in detail the pathways that potentially explain how lung inflammation can trigger acute vascular events such as heart attacks and stroke. Lung inflammation due to COPD, asthma, infection, or exposure to air pollution results in a systemic inflammatory response (split over) with increase in the levels of circulating leukocytes, platelets, cytokines, and acute-phase proteins. These mediators activate the vascular endothelium, causing endothelial dysfunction that is characterized by reduced vasodilatation with decreases in nitric oxide (NO), increases in endothelin (ET) expression, and increases in vascular permeability and the uptake of oxidized low-density lipoproteins (LDLs) into atherosclerotic plaques. Collectively, these events destabilize plaque by the up-regulation of adhesion molecules with accelerated leukocyte recruitment, increase foam cell formation and the recruitment of smooth muscle cells, release and activate proteases that degrade the extracellular matrix and destabilize plaques, making them vulnerable for rupture [122]. Indeed, a cohort study demonstrated the close interplay between systemic endothelial dysfunction and lung dysfunction could begin already prior to the development of overt respiratory or cardiovascular disease and suggest that even individuals with mild impairment of lung function may have vascular damage that increase the risk for cardiovascular disease [123]. Therefore, patients with asthma may induce an inflammatory environment that favor atherosclerosis progression [124].

The burden of cardiovascular comorbidity in obstructive airway disease is increasingly acknowledged, and there is a need of identifying which patients are at an increased risk and thus to facilitate optimal treatment and prevention [125].

### Impact of stroke in lung function

Pulmonary complications, such as respiratory failure, pneumonia, pleural effusion, acute respiratory distress syndrome, pulmonary edema, and pulmonary embolism from venous thromboembolism, are common in stroke and are among the major causes of death in stroke patients [126]. For instance, a cohort study assessed the lung function of stroke survivors and the main finding was that lung function was significantly lower in stroke patients compared with healthy participants: lower values for FEV1 (81% of predicted value vs. 95% predicted), FVC (82% vs 92% of predicted values), and PEF (52% vs 70%). Also, chest excursion was markedly lower for stroke survivors when compared to control group (3.0 ± 0.71 vs 3.5 ± 0.91 cm), which may result from weakened respiratory muscles [127].

Besides, Jung and coworkers demonstrated through ultrasonographic diaphragmatic motion analysis that diaphragmatic excursion in right-hemiplegic patients was reduced on both sides compared to that in control subjects. However, in left-hemiplegic patients diaphragmatic excursion is reduced on the left side and increased on the right side compared to that in control subjects and left diaphragmatic motion during deep breathing correlates positively with FVC (r = 0.86, *p* = 0.007) and FEV1 (r = 0.79, *p* = 0.021) [128].

The mechanism of lung damage after brain injury is described through a “double hit model”: the catecholamine storm and the systemic production of inflammatory mediators (first hit) create a systemic inflammatory environment which increases pulmonary vascular hydrostatic pressure and activates biological mechanisms that make the lung more susceptible to mechanical and non-mechanical insults (second hit), including mechanical ventilation. Indeed, a study on mice showed that ischemic stroke caused a significant increase in bronchoalveolar lavage fluid macrophages and neutrophils and whole lung tissue pro-inflammatory IL-1βmRNA expression [129]. Furthermore, the phagocytic ability of macrophages from BALF is markedly reduced in post-stroke rats [130]. Thus, damage to the alveolar capillary barrier leads to pulmonary ventilation disorder, blood perfusion disorder and oxygenation disorder, such as acute, life-threatening neurogenic pulmonary edema that occurs in about 23% of SAH patients [131].

After stroke, damage to the blood-brain barrier leads to recruitment of resident and peripheral immune cells to the affected area, resulting in a reduction in circulating immune cells and a depression of peripheral immunity that increases the susceptibility to infection [126]. Indeed, a study on mice revealed that immunosuppression after stroke is related to an increased expression of inflammatory mediators and hypothalamic-pituitary-adrenal axis activation which induces elevated glucocorticoid secretion [132].

Stroke-associated pneumonia incidence is high and can be due to: stroke-induced immunodepression syndrome, dysphagia, decreased level of consciousness, all risk factors for aspiration pneumonia [126]. In fact, a cohort study demonstrated that in ischemic stroke patients requiring invasive ventilation, pneumonia occurred in 40% of cases and was associated with a 49% increase in 1-year mortality [133].

Thus, brain-lung crosstalk is relevant to prevent further pulmonary complications after stroke. In detail, protective ventilation has to be considered in this population to obtain the target of normoxia and normocapnia avoiding high tidal volume. Respiratory muscle training showed to improve the strength and decrease the risk of respiratory complications in stroke survivors [134]. Also, interventions targeting plasma fibronectin may reduce brain damage following reperfusion – a promising reperfusion therapy for patients with acute ischemic stroke [135].

### Impact of stroke on asthma outcomes

Stroke is associated with major complications, such as, dysphagia, GERD, aspiration, immunodepression and pneumonia [136, 137]. Obstructive airway disease, such as asthma, is the most common extraesophageal manifestation of GERD, with a prevalence of 52.67% in a cohort study and a statistically significant correlation of severity of GERD and severity of bronchial asthma [138]. In addition, GERD is responsible for a high number of annual exacerbations, consultations, hospitalizations and very frequent use of short-acting bronchodilators in asthma patients [139].

Besides, GERD and the use of acid suppressing agents (histamine-2 receptor antagonists and proton pump inhibitors) which are commonly used in GERD [140]; dysphagia, the compromised immune state and the use of corticosteroids are all risk factors for SAP [141]. SAP is not associated with increased long-term mortality, but it is linked with increased mortality up to 1 y, prolonged length of stay, and poor functional outcome on discharge [142].

### Impact of asthma on stroke outcomes

Stroke outcomes could be influenced in long-term by chronic inflammatory airway disease. In a cohort study, history of CIAD was independently associated with mortality during long-term follow-up (HR = 1.42, 95% CI = 1.02–2.00). However, CIAD was not significantly associated with short-term mortality after stroke. Furthermore, CIAD is an independent risk factor for pneumonia after stroke – pneumonia being a major cause of death in stroke patients [143]. Also, there is a consistent, independent and long-lasting association between lung function and fatal stroke [34]. A statistical interplay between mortality due to stroke in asthma patients was demonstrated by Strand and coworkers. Moreover, individuals with active asthma showed an increased risk of dying from CVD [38]. Stroke outcomes might be exacerbated by fibronectin, which promotes inflammation of the thrombus [135].

As we underlined previously, that asthma patients are at risk in developing atherosclerosis, atrial fibrillation, myocardial infarction, stroke and maybe even recurrent stroke. Asthma exacerbations, ACO and uncontrolled asthma could have an additional risk for poorer prognosis after stroke. Further studies should investigate whether the incidence of worse outcomes after stroke and recurrent stroke in asthma patients is increased. Similarly, in the current guidelines, asthma is not considered a relevant comorbidity to be addressed for primary or secondary stroke prevention. However, current data reveals a relevant interaction between asthma and stroke and thus this statement should be reviewed.

### Prevention of stroke in asthma patients

Since stroke is an acute, burdensome, and preventable condition several preventive options may be useful in asthma patients.

Firstly, we should focus on modifiable risk factors for stroke, such as obesity and tobacco use, that could certainly have a great impact on asthma outcomes, stroke prevention, as well as, stroke outcomes. Obesity can trigger asthma development through several mechanism, but also it is associated with worsening of asthma symptoms, increased exacerbations, unresponsiveness to standard therapy [144]. Scott and coworkers found that weight loss in an obese asthma population significant improves health status among participants [145]. In addition, there is evidence that obesity is associated with bronchodilator unresponsiveness among black and Latino children and adolescents with asthma [146]. Obesity-related asthma which usually develops in adulthood might be a particular importance to the development of CVD events [38]. The importance of smoking cessation we have elucidated so far.

As we emphasized the importance of monitoring asthma patients via standard clinical and laboratory tests, in order to identify subclinical atherosclerosis prior to progression to full CVD complicated by acute events. We mentioned the acute phase reactants, like CRP, hepcidin, fibrinogen; fibronectin, α2-macroglobulin, PAI-1, von Willebrand factor, D-dimer, brain natriuretic peptide; measurement of clot lysis time, arterial stiffness through PWV. All of these are either detecting and thus preventing CVD events or are predictors of the outcomes from those events. A study suggested that routine administration of the CVHI in a primary prevention population would yield the benefits of identifying patients with existing subclinical CVD [147].

Asthmatics have a prothrombotic state that could be counteracted by heparin or enoxaparin therapy. Early studies described subjective improvement of asthma symptoms using intravenous or inhaled heparin [148]. Especially for asthma patients with estimated CVD and stroke risk it should be taken into account the adjuvant anticoagulant therapy. Furthermore, clinical studies of patients with asthma reveal heightened platelet activation and accumulation into lung tissue, thus suggesting the need for further research to exploit the potentially powerful anti-inflammatory applications possessed by anti-platelet drugs [149].

In addition, the therapy of each asthma patient should be customized according to its severity, phenotype and endotype, its congruency and response to it, as well as current comorbidities. Target therapy is the future strategy, that could minimize the risk for complications, such as stroke [83].

Statins have been shown to have multiple pleiotropic effects other than its lipid lowering activity by modulating multiple signaling pathways that govern inflammatory, mucus-inhibitory, oxidant stress and proliferation. Thus, the repurposing of statins from conventional anti-cholesterol oral therapy to inhaled anti-inflammatory formulation is promising, as it provides direct delivery to the airways, reduced risk of side effects, increased bioavailability and tailored physical-chemical properties for enhanced efficacy. Inhaled statins act by reducing airway inflammation and oxidation; regulating NOS, as well as, attenuating airway remodeling by regulation of MMP expressions and decrease MUC gene expression [150]. Indeed, a cohort study demonstrated that CHD risk was lower in all statin users, regardless of the duration of use, whereas ischemic stroke risk was lower only in the long-term statin users [24]. Consistent with these results, Chou et al. reported that in adults with a high risk of CVD but no prior CVD events, statin use is associated with a low risk of CVD events, and patients at a high baseline risk have relatively greater absolute benefits (e.g., those with hypercholesterolemia) [151]. Moreover, the use of ICS or OS with statins has an additive effect [152]. A group showed that treatment of OVA-exposed mice with i.t. pravastatin (30 mg/kg) improved asthma pathology [153]. Oxidative stress is a constantly discussed component of both of the disease and there is ongoing research for adequate antioxidant therapy [154].

Vitamin D is a recognized modulator of the immune response, which is required for an adequate physiologic response to inflammatory diseases and immune-system mediated diseases; thus, vitamin D status play a role in the association between asthma and stroke. Indeed, calcitriol acts as a direct transcriptional regulator of endothelial nitric oxide (NO) synthase (eNOS), and can promote normalization of eNOS mRNA expression and enzymatic activity in experimental atherosclerosis. Overall, it is plausible that the impaired endothelial function that may accompany low circulating vitamin D levels contributes to an increased risk of cerebrovascular diseases and mortality [155]. In asthma, reduced vitamin D levels are associated with impaired lung function, increased AHR, and reduced GC response, suggesting that supplementation of vitamin D levels in patients with asthma may improve a number 0f parameters of asthma severity and treatment response [156]. Actually, a cohort study revealed that FEV1 percent predicted and FEV1/forced vital capacity ratio showed a significant positive correlation with vitamin D levels. Also, the use of inhaled steroids, use of oral steroids, and the total steroid dose all showed significant inverse correlations with vitamin D levels. This findings support that vitamin D enhances the anti-inflammatory effects of glucocorticoids, and could be as a potential steroid-sparing agent in patients with moderate-to-severe persistent asthma, as well as a modifier of asthma disease severity [157]. Furthermore, low serum levels of vitamin D at admission have been proposed as an independent prognostic biomarker for greater stroke severity, a poorer functional outcome at discharge, a higher risk of death at one or 2 y, and a greater risk of early recurrent stroke [158, 159].

Vagal nerve stimulation appears to be a safe and feasible modality for use in the treatment of moderate to severe, acute asthma exacerbations in patients unresponsive to initial standard of care and as a rescue intervention for mild-to-moderate asthmatic attacks. Maybe neurostimulation could be an option for management of asthma exacerbations, thus preventing complications [160].

## Summary and future perspective

**Fig. 3:**
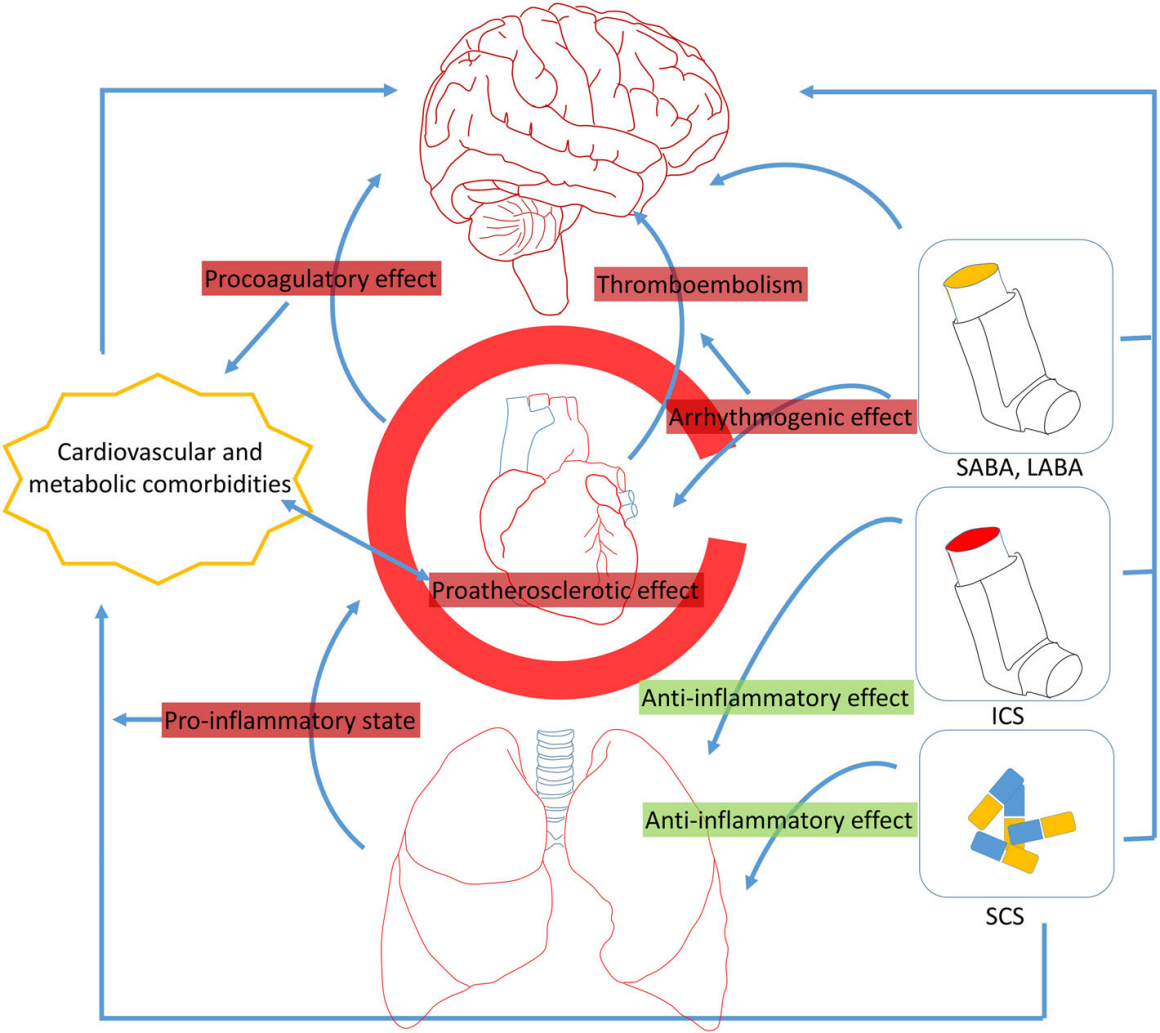
The complex interaction between lungs and the brain

It is important to clarify whether asthma increase the risk of all stroke types and to elaborate safe and effective methods for stroke prevention. More studies are required to evaluate whether, adjusting the therapy with anticoagulants is a correct strategy, as well as, which predictors are the best to be implemented in routine monitoring of asthma patients. In general, the interaction between lungs and the brain is complex (Fig. 3). The pro-inflammatory state generated at the level of the lungs leads to atherosclerosis, comorbid conditions, procoagulatory state. SCS therapy decreases the inflammatory process but in turn, also leads to cardiovascular and metabolic diseases. Although, ICS are safer, they are also linked to comorbidities. SABA and LABA are directly linked to arythmogenic effect, which in turn can lead to thromboembolism. It seems that all of the abovementioned main drug groups that are used for asthma treatment are to some degree linked to stroke. Antimuscarinic agents on the other did not demonstrate this effect. Similarly, there are other emerging drug groups which may demonstrate a better safety profile regarding cardiovascular and metabolic comorbidities as well as stroke risk. We also need agents that can effectively combat and limit the inflammatory state at the level of the lungs. Asthma-related comorbidities should be at special attention in lights of the fact that the majority are linked to cerebrovascular disease.

No clinical studies were found on the use of inhaled statins. Furthermore, studies on reformulating statins as an inhaled therapy are still in their infancy and further investigations are required to better understand the efficacy, toxicity and mechanism of action of these statin molecules in the airways and in the prevention of stroke.

The findings of vitamin D implications in asthma evolution and stroke outcomes should be confirmed in a prospective fashion that involves the generation of an efficient multivariate model, in order to include vitamin D supplementation as an adjuvant therapy for asthma patients, especially those with increased risk for stroke.

Furthermore, current treatment options are limited and may not be effective for all patient populations. Hence, new treatment options with superior efficacies to treat these diseases are urgently required as potential substitution, alternative or adjunct therapy to currently available therapies.

## Conclusions

Asthma is a heterogeneous disease with several key pathophysiological mechanisms that impact the whole body. There is enough data that suggests the association between asthma and atherosclerosis, which in turn leads to CVD and stroke. It seems that asthma may increase the risk of both ischemic and hemorrhagic stroke. The proper management of asthma, prevention of exacerbations, as well as, prospective monitoring and the use of adjuvant therapy are essential to decrease the risk for stroke and improve its outcomes. Asthma comorbidities should be at special attention and there is need for a better understanding of limitations of the current treatment strategies.

## Data Availability

Not applicable.

## Abbreviations

ACO: Asthma-COPD overlap
AHR: Airway hyperresponsiveness
TPA: Tissue plasminogen activator
baPWV: Brachial-ankle pulse wave velocity
BALF: Bronchoalveolar lavage fluid
BMI: Body mass index
CIMT: Carotid intima media thickness
CVD: Cardiovascular disease
CHD: Coronary heart disease
CVHI: Cardiovascular health index
CIAD: Chronic inflammatory airway disease
COPD: Chronic obstructive pulmonary disease
CRP: Reactive C protein
ECP: Eosinophilic cation protein
HR: Hazard ratio
GC: Glucocorticoids
GERD: Gastroesophageal reflux disease
ICS: Inhaled corticosteroids
IL: Interleukin
PAI-1: Plasminogen activator inhibitor 1
PF-4: Platelet factor 4
TNFα: Tumor necrosis factor alfa
FEV1: Forced expiratory volume in 1 s
FIMT: Femoral intima media thickness
SABA: Short acting beta agonists
SAH: Subarachnoid hemorrhage
SAP: Stroke induced pneumonia
SCS: Systemic corticosteroids
LABA: Long acting beta agonists
LAMA: Long acting muscarinic antagonists
NOS: Nitric oxide synthase
MUC: Mucin gene
MMP: Matrix metalloproteinase

## References

[1] (GINA) Global Strategy for Asthma Management and Prevention. 2020. Available from: https://www.ginasthma.org. Accessed 20 Dec 2020.

[2] Covantev S, Mazuruc N, Uzdenov R, Corlateanu A (2019). Spontaneous Pneumomediastinum &ndash; a Rare Asthma Complication. Folia Med.

[3] Cepelis A, Brumpton BM, Laugsand LE, Langhammer A, Janszky I, Strand LB. Asthma, asthma control and risk of ischemic stroke: The HUNT study. Respir Med. 2020 2020/11/01/;2:100013.

[4] Raita Y, Camargo CA, Faridi MK, Brown DFM, Shimada YJ, Hasegawa K. Risk of Acute Myocardial Infarction and Ischemic Stroke in Patients with Asthma Exacerbation: A Population-Based, Self-Controlled Case Series Study. J Allerg Clin Immunol. 2020 2020/01/01/;8(1):188–194. e8. 10.1016/j.jaip.2019.06.043 31323338

[5] Zeiger R, Sullivan P, Chung Y, Kreindler JL, Zimmerman NM, Tkacz J. Systemic Corticosteroid-Related Complications and Costs in Adults with Persistent Asthma. J Allerg Clin Immunol. 2020 2020/11/01/; 8(10):3455–3465.e13. 10.1016/j.jaip.2020.06.055 32679349

[6] Cazzola M, Rogliani P, Calzetta L, Matera MG. Bronchodilators in subjects with asthma-related comorbidities. Respir Med. 2019 2019/05/01/;151: 43–48. 10.1016/j.rmed.2019.04.001 31047116

[7] Tuleta I, Skowasch D, Aurich F, Eckstein N, Schueler R, Pizarro C (2017). Asthma is associated with atherosclerotic artery changes. PLoS One.

[8] Katan M, Luft A (2018). Global burden of stroke. Semin Neurol.

[9] Corlateanu A, Covantev S, Mathioudakis AG, Botnaru V, Cazzola M, Siafakas N. Chronic obstructive pulmonary disease and stroke. Copd. 2018;15(4):405–413. PubMed PMID: 29746193. Epub 2018/05/11. eng. 10.1080/15412555.2018.1464551 29746193

[10] Chung WS, Lin CL, Chen YF, Ho FM, Hsu WH, Kao CH (2014). Increased stroke risk among adult asthmatic patients. Eur J Clin Investig.

[11] Balofsky A, George J, Papadakos P. Chapter 3 - Neuropulmonology. In: Wijdicks EFM, Kramer AH, editors. Handbook of Clinical Neurology. 140: Elsevier; 2017. p. 33–48. 10.1016/B978-0-444-63600-3.00003-9 28187807

[12] Ma S, Zhao H, Ji X, Luo Y. Peripheral to central: Organ interactions in stroke pathophysiology. Exp Neurol. 2015 2015/10/01/; 272:41–49. 10.1016/j.expneurol.2015.05.014 26054885

[13] Mai X, Liang X. Risk Factors for Stroke Based on the National Health and Nutrition Examination Survey. J Nutr Health Aging. 2020 2020/07/01; 24(7):791–795.10.1007/s12603-020-1430-432744577

[14] Kim SY, Lim H, Lim J-S, Choi HG. Analysis of the Relationship between Adult Asthma and Stroke: A Longitudinal Follow-Up Study Using the Korean National Sample Cohort. BioMed Res Int. 2019 2019/06/17;2019:8919230. 10.1155/2019/8919230 PMC6601683 31317041

[15] Wen L-Y, Ni H, Li K-S, Yang H-H, Cheng J, Wang X, et al. Asthma and Risk of Stroke: A Systematic Review and Meta-analysis. J Stroke Cerebrovasc Dis. 2016 2016/03/01/;25(3):497–503. 10.1016/j.jstrokecerebrovasdis.2015.11.030 26803721

[16] Rodrigo C, Rodrigo G. Subarachnoid hemorrhage following permissive hypercapnia in a patient with severe acute asthma. Am J Emerg Med 1999;17(7):697–699. PubMed PMID: 10597094. Epub 1999/12/22. eng. 10.1016/s0735-6757(99)90164-x 10597094

[17] Edmunds SM, Harrison R. Subarachnoid hemorrhage in a child with status asthmaticus: significance of permissive hypercapnia. Pediatr Crit Care Med. 2003 2003/01//;4(1):100–103. PubMed PMID: 12656553. eng. 10.1097/00130478-200301000-00020 12656553

[18] Söderholm M, Zia E, Hedblad B, Engström G. Lung function as a risk factor for subarachnoid hemorrhage: a prospective cohort study. Stroke. 2012;43(10):2598–2603. PubMed PMID: 22871680. Epub 2012/08/09. eng. 10.1161/STROKEAHA.112.658427 22871680

[19] Hankey GJ (2020). Population impact of potentially modifiable risk factors for stroke. Stroke..

[20] Yousuf H, Hofstra M, Tijssen J, Leenen B, Lindemans JW, van Rossum A (2020). Estimated Worldwide Mortality Attributed to Secondhand Tobacco Smoke Exposure, 1990–2016. JAMA Netw Open.

[21] Woodward A, Laugesen M (2001). How many deaths are caused by second hand cigarette smoke?. Tob Control.

[22] Çolak Y, Afzal S, Nordestgaard BG, Lange P (2015). Characteristics and prognosis of never-smokers and smokers with asthma in the Copenhagen general population study. A prospective cohort study. Am J Respir Crit Care Med.

[23] Portegies MLP, Lahousse L, Joos GF, Hofman A, Koudstaal PJ, Stricker BH (2016). Chronic obstructive pulmonary disease and the risk of stroke. The Rotterdam study. Am J Respir Crit Care Med.

[24] Yeh J-J, Lin C-L, Hsu CY, Shae Z, Kao C-H. Associations between statins and coronary artery disease and stroke risks in patients with asthma–chronic obstructive pulmonary disease overlap syndrome: A time-dependent regression study. Atherosclerosis. 2019 2019/04/01/; 283:61–68. 10.1016/j.atherosclerosis.2019.02.007 30782562

[25] Vozoris NT, Stanbrook MB (2011). Smoking prevalence, behaviours, and cessation among individuals with COPD or asthma. Respir Med.

[26] W-x S, Jin D, Li Y, Wang R-t (2014). Increased arterial stiffness in stable and severe asthma. Respir Med.

[27] Yılmaz M, Bozkurt Yılmaz HE, Şen N, Altın C, Tekin A, Müderrisoğlu H. Investigation of the relationship between asthma and subclinical atherosclerosis by carotid/femoral intima media and epicardial fat thickness measurement. J Asthma. 2018 2018/01/02;55(1):50–56. 10.1080/02770903.2017.1313272 28453377

[28] Bazan-Socha S, Mastalerz L, Cybulska A, Zareba L, Kremers R, Zabczyk M (2016). Asthma is associated with enhanced thrombin formation and impaired fibrinolysis. Clin Exp Allergy.

[29] Bazan-Socha S, Mastalerz L, Cybulska A, Zareba L, Kremers R, Zabczyk M, et al. Prothrombotic State in Asthma Is Related to Increased Levels of Inflammatory Cytokines, IL-6 and TNFα, in Peripheral Blood. Inflammation. 2017 2017/08/01;40(4):1225–1235. 10.1007/s10753-017-0565-x PMC5494034 28429138

[30] Sneeboer MMS, Majoor CJ, de Kievit A, Meijers JCM, van der Poll T, Kamphuisen PW, et al. Prothrombotic state in patients with severe and prednisolone-dependent asthma. J Allerg Clin Immunol. 2016 2016/06/01/; 137(6):1727–1732. 10.1016/j.jaci.2015.10.038 26714414

[31] Niccoli G, Ferrante G, Cosentino N, Conte M, Belloni F, Marino M (2010). Eosinophil cationic protein: a new biomarker of coronary atherosclerosis. Atherosclerosis..

[32] Kuczia P, Mastalerz L, Potaczek DP, Cybulska A, Zareba L, Bazan-Socha S, et al. Increased activity of lipoprotein-associated phospholipase A2 in non-severe asthma. Allergol Int. 2019 2019/10/01/; 68(4):450–455. 10.1016/j.alit.2019.04.004 31064688

[33] Bazan-Socha S, Kuczia P, Potaczek DP, Mastalerz L, Cybulska A, Zareba L, et al. Increased blood levels of cellular fibronectin in asthma: Relation to the asthma severity, inflammation, and prothrombotic blood alterations. Respir Med. 2018 2018/08/01/; 141:64–71. 10.1016/j.rmed.2018.06.023 30053974

[34] Gulsvik AK, Gulsvik A, Skovlund E, Thelle DS, Mowé M, Humerfelt S (2012). The association between lung function and fatal stroke in a community followed for 4 decades. J Epidemiol Community Health.

[35] Hozawa A, Billings JL, Shahar E, Ohira T, Rosamond WD, Folsom AR. Lung function and ischemic stroke incidence: the atherosclerosis risk in communities study. Chest. 2006;130(6):1642–1649. PubMed PMID: 17166977. Epub 2006/12/15. eng. 10.1378/chest.130.6.1642 17166977

[36] Weiler Z, Zeldin Y, Magen E, Zamir D, Kidon MI. Pulmonary function correlates with arterial stiffness in asthmatic patients. Respir Med. 2010 2010/02/01/; 104(2): 197–203. 10.1016/j.rmed.2009.09.004 19892539

[37] Tattersall MC, Guo M, Korcarz CE, Gepner AD, Kaufman JD, Liu KJ (2015). Asthma predicts cardiovascular disease events: the multi-ethnic study of atherosclerosis. Arterioscler Thromb Vasc Biol.

[38] Strand LB, Tsai MK, Wen CP, Chang SS, Brumpton BM (2018). Is having asthma associated with an increased risk of dying from cardiovascular disease? A prospective cohort study of 446 346 Taiwanese adults. BMJ Open.

[39] Wee JH, Park MW, Min C, Byun SH, Park B, Choi HG (2020). Association between asthma and cardiovascular disease. Eur J Clin Invest..

[40] Cepelis A, Brumpton BM, Malmo V, Laugsand LE, Loennechen JP, Ellekjær H (2018). Associations of asthma and asthma control with atrial fibrillation risk: results from the Nord-Trøndelag health study (HUNT). JAMA Cardiol.

[41] Lewis MJ, Short AL, Lewis KE (2006). Autonomic nervous system control of the cardiovascular and respiratory systems in asthma. Respir Med.

[42] Iribarren C, Tolstykh IV, Miller MK, Sobel E, Eisner MD (2012). Adult asthma and risk of coronary heart disease, cerebrovascular disease, and heart failure: a prospective study of 2 matched cohorts. Am J Epidemiol.

[43] Onufrak S, Abramson J, Vaccarino V. Adult-onset asthma is associated with increased carotid atherosclerosis among women in the atherosclerosis risk in communities (ARIC) study. Atherosclerosis. 2007 2007/11/01/; 195(1):129–137. 10.1016/j.atherosclerosis.2006.09.004 PMC2128256 17045272

[44] Hekking P-P, Loza MJ, Pavlidis S, de Meulder B, Lefaudeux D, Baribaud F, et al. Pathway discovery using transcriptomic profiles in adult-onset severe asthma. J Allergy Clin Immunol. 2018 2018/04/01/; 141(4): 1280–1290. 10.1016/j.jaci.2017.06.037 28756296

[45] de Nijs SB, Venekamp LN, Bel EH (2013). Adult-onset asthma: is it really different?. Eur Respir Rev.

[46] Nyenhuis SM, Akkoyun E, Liu L, Schatz M, Casale TB. Real-World Assessment of Asthma Control and Severity in Children, Adolescents, and Adults with Asthma: Relationships to Care Settings and Comorbidities. J Allergy Clin Immunol. 2020 2020/03/01/; 8(3):989–996.e1. 10.1016/j.jaip.2019.10.032 PMC7064399 31707065

[47] Schanen JG, Iribarren C, Shahar E, Punjabi NM, Rich SS, Sorlie PD, et al. Asthma and incident cardiovascular disease: the atherosclerosis risk in communities study. Thorax. 2005 Aug;60(8):633–638. PubMed PMID: 16061703. Pubmed Central PMCID: PMC1747501. Epub 2005/08/03. eng. 10.1136/thx.2004.026484 PMC1747501 16061703

[48] Bazan-Socha S, Mastalerz L, Cybulska A, Zareba L, Kremers R, Zabczyk M, et al. Impaired fibrinolysis and lower levels of plasma α2-macroglobulin are associated with an increased risk of severe asthma exacerbations. Sci Rep. 2017 2017/09/08; 7 (1): 11014. 10.1038/s41598-017-11467-8 PMC5591306 28887505

[49] Corlateanu A, Covantev S, Mathioudakis AG, Botnaru V, Siafakas N. Ashtma-Chronic obstructive pulmonary disease overlap syndrome (ACOS): current evidence and future research directions. COPD Res Pract. 2017 2017/05/30; 3(1):6.

[50] Llanos J-P, Ortega H, Germain G, Duh MS, Lafeuille M-H, Tiggelaar S, et al. Health characteristics of patients with asthma, COPD and asthma-COPD overlap in the NHANES database. Int J Chron Obstruct Pulmon Dis. 2018; 13: 2859–2868. PubMed PMID: 30254433. eng. 10.2147/COPD.S167379 PMC6143639 30254433

[51] Yeh JJ, Wei YF, Lin CL, Hsu WH (2017). Effect of the asthma-chronic obstructive pulmonary disease syndrome on the stroke, Parkinson's disease, and dementia: a national cohort study. Oncotarget..

[52] Corlateanu A, Covantev S, Mathioudakis AG, Botnaru V, Siafakas N. Prevalence and burden of comorbidities in chronic obstructive pulmonary disease. Respir Investig 2016;54(6):387–396. PubMed PMID: 27886849. Epub 2016/11/26. eng. 10.1016/j.resinv.2016.07.001 27886849

[53] Boulet L-P, Boulay M-È. Asthma-related comorbidities. Expert Rev Respir Med. 2011 2011/06/01; 5(3):377–393. 10.1586/ers.11.34 21702660

[54] Kankaanranta H, Kauppi P, Tuomisto LE, Ilmarinen P. Emerging Comorbidities in Adult Asthma: Risks, Clinical Associations, and Mechanisms. Mediat Inflamm. 2016 2016/04/26; 3690628. 10.1155/2016/3690628 PMC4861800 27212806

[55] Tsai C-L, Lee W-Y, Hanania NA, Camargo Jr CA. Age-related differences in clinical outcomes for acute asthma in the United States, 2006–2008. J Allerg Clin Immunol. 2012; 129(5):1252–1258. e1. 10.1016/j.jaci.2012.01.061 22385630

[56] Li C, Engström G, Hedblad B, Berglund G, Janzon L. Blood pressure control and risk of stroke: a population-based prospective cohort study. Stroke. 2005;36(4):725–730. PubMed PMID: 15746450. Epub 2005/03/05. eng. 10.1161/01.STR.0000158925.12740.87 15746450

[57] Weatherburn CJ, Guthrie B, Mercer SW, Morales DR (2017). Comorbidities in adults with asthma: population-based cross-sectional analysis of 1.4 million adults in Scotland. Clin Exp Allergy.

[58] Panek M, Mokros Ł, Pietras T, Kuna P. The epidemiology of asthma and its comorbidities in Poland – Health problems of patients with severe asthma as evidenced in the Province of Lodz. Respir Med. 2016 2016/03/01/; 112:31–38. 10.1016/j.rmed.2016.01.009 26852088

[59] Sobiczewski W, Wirtwein M, Trybala E, Gruchala M. Severity of coronary atherosclerosis and stroke incidence in 7-year follow-up. J Neurol. 2013; 260(7):1855–1858. PubMed PMID: 23512577. Epub 03/20. eng. 10.1007/s00415-013-6892-4 PMC3705141 23512577

[60] Taha M, Mishra T, Shokr M, Sharma A, Taha M, Samavati L (2020). Burden and impact of arrhythmias in asthma-related hospitalizations: Insight from the national inpatient sample. J Arrhythmia..

[61] Cepelis A, Brumpton BM, Malmo V, Laugsand LE, Loennechen JP, Ellekjær H, et al. Associations of asthma and asthma control with atrial fibrillation risk: results from the Nord-Trøndelag health study (HUNT). JAMA Cardiol. 2018; 3(8): 721-728. PubMed PMID: 29998294. Pubmed central PMCID: PMC6143075 form for disclosure of potential conflicts of interest. No disclosures were reported Epub 2018/07/13. eng. 10.1001/jamacardio.2018.1901 PMC6143075 29998294

[62] Go AS, Reynolds K, Yang J, Gupta N, Lenane J, Sung SH (2018). Association of burden of atrial fibrillation with risk of ischemic stroke in adults with paroxysmal atrial fibrillation: the KP-RHYTHM study. JAMA Cardiol.

[63] Gibeon D, Batuwita K, Osmond M, Heaney LG, Brightling CE, Niven R, et al. Obesity-associated severe asthma represents a distinct clinical phenotype: analysis of the British Thoracic Society difficult asthma registry patient cohort according to BMI. Chest. 2013;143(2):406–414. PubMed PMID: 23064546. Epub 2012/10/16. eng. 10.1378/chest.12-0872 23064546

[64] Moore WC, Meyers DA, Wenzel SE, Teague WG, Li H, Li X, et al. Identification of asthma phenotypes using cluster analysis in the severe asthma research program. Am J Respir Crit Care Med 2010;181(4):315–323. PubMed PMID: 19892860. Pubmed Central PMCID: PMC2822971. Epub 2009/11/07. eng. 10.1164/rccm.200906-0896OC PMC2822971 19892860

[65] Shaw DE, Sousa AR, Fowler SJ, Fleming LJ, Roberts G, Corfield J, et al. Clinical and inflammatory characteristics of the European U-BIOPRED adult severe asthma cohort. Eur Respir J 2015;46(5):1308–1321. PubMed PMID: 26357963. Epub 2015/09/12. eng. 10.1183/13993003.00779-2015 26357963

[66] van Veen IH, Ten Brinke A, Sterk PJ, Rabe KF, Bel EH. Airway inflammation in obese and nonobese patients with difficult-to-treat asthma. Allergy. 2008;63(5):570–574. PubMed PMID: 18394131. Epub 2008/04/09. eng. 10.1111/j.1398-9995.2007.01597.x 18394131

[67] Mitchell AB, Cole JW, McArdle PF, Cheng Y-C, Ryan KA, Sparks MJ, et al. Obesity increases risk of ischemic stroke in young adults. Stroke. 2015;46(6):1690–1692. PubMed PMID: 25944320. Epub 05/05. eng. 10.1161/STROKEAHA.115.008940 PMC4458137 25944320

[68] Cazzola M, Calzetta L, Bettoncelli G, Novelli L, Cricelli C, Rogliani P (2011). Asthma and comorbid medical illness. Eur Respir J.

[69] Shah R, Yang Y. Health and economic burden of obesity in elderly individuals with asthma in the United States. Popul Health Manag 2015;18(3):186–191. PubMed PMID: 25291085. Epub 2014/10/08. eng. 10.1089/pop.2014.0089 25291085

[70] Auckley D, Moallem M, Shaman Z, Mustafa M. Findings of a Berlin questionnaire survey: comparison between patients seen in an asthma clinic versus internal medicine clinic. Sleep Med 2008;9(5):494–499. PubMed PMID: 17766180. Epub 2007/09/04. eng. 10.1016/j.sleep.2007.06.010 17766180

[71] Julien JY, Martin JG, Ernst P, Olivenstein R, Hamid Q, Lemière C, et al. Prevalence of obstructive sleep apnea-hypopnea in severe versus moderate asthma. J Allergy Clin Immunol 2009;124(2):371–376. PubMed PMID: 19560194. Epub 2009/06/30. eng. 10.1016/j.jaci.2009.05.016 19560194

[72] Munoz R, Duran-Cantolla J, Martínez-Vila E, Gallego J, Rubio R, Aizpuru F (2006). Severe sleep apnea and risk of ischemic stroke in the elderly. Stroke..

[73] Bor S, Kitapcioglu G, Solak ZA, Ertilav M, Erdinc M. Prevalence of gastroesophageal reflux disease in patients with asthma and chronic obstructive pulmonary disease. J Gastroenterol Hepatol 2010 Feb;25(2):309–313. PubMed PMID: 19817951. Epub 2009/10/13. eng. 10.1111/j.1440-1746.2009.06035.x 19817951

[74] Harding SM. The potential role of gastroesophageal reflux in asthma. Minerva Gastroenterol Dietol 2001;47(2):75–83. PubMed PMID: 16493363. Epub 2006/02/24. eng. 16493363

[75] Sheu JJ, Kang JH, Lou HY, Lin HC. Reflux esophagitis and the risk of stroke in young adults: a 1-year population-based follow-up study. Stroke. 2010;41(9):2033–2037. PubMed PMID: 20651264. Epub 2010/07/24. eng. 10.1161/STROKEAHA.110.588558 20651264

[76] Otsuki M, Miyatake A, Fujita K, Hamasaki T, Kasayama S (2010). Reduced carotid atherosclerosis in asthmatic patients treated with inhaled corticosteroids. Eur Respir J.

[77] Varas-Lorenzo C, Rodriguez LAG, Maguire A, Castellsague J, Perez-Gutthann S (2007). Use of oral corticosteroids and the risk of acute myocardial infarction. Atherosclerosis..

[78] Daugherty J, Lin X, Baxter R, Suruki R, Bradford E (2017). The impact of long-term systemic glucocorticoid use in severe asthma: a UK retrospective cohort analysis. J Asthma.

[79] Grayson MH, Feldman S, Prince BT, Patel PJ, Matsui EC, Apter AJ. Advances in asthma in 2017: Mechanisms, biologics, and genetics. J Allerg Clin Immunol. 2018 2018/11/01/; 142(5): 1423–1436. 10.1016/j.jaci.2018.08.033 30213625

[80] Nair P, Wenzel S, Rabe KF, Bourdin A, Lugogo NL, Kuna P (2017). Oral glucocorticoid–sparing effect of Benralizumab in severe asthma. N Engl J Med.

[81] Wenzel S, Castro M, Corren J, Maspero J, Wang L, Zhang B, et al. Dupilumab efficacy and safety in adults with uncontrolled persistent asthma despite use of medium-to-high-dose inhaled corticosteroids plus a long-acting β2 agonist: a randomised double-blind placebo-controlled pivotal phase 2b dose-ranging trial. Lancet. 2016 2016/07/02/; 388(10039):31–44. 10.1016/S0140-6736(16)30307-5 27130691

[82] Corren J, Parnes JR, Wang L, Mo M, Roseti SL, Griffiths JM (2017). Tezepelumab in adults with uncontrolled asthma. N Engl J Med.

[83] Menzella F, Bertolini F, Biava M, Galeone C, Scelfo C, Caminati M. Severe refractory asthma: current treatment options and ongoing research. Drugs Context. 2018;7:212561. PubMed PMID: 30534175. eng. 10.7573/dic.212561 PMC6284776 30534175

[84] Iribarren C, Rahmaoui A, Long AA, Szefler SJ, Bradley MS, Carrigan G, et al. Cardiovascular and cerebrovascular events among patients receiving omalizumab: Results from EXCELS, a prospective cohort study in moderate to severe asthma. J Allerg Clin Immunol. 2017 2017/05/01/;139(5): 1489–1495.e5. 10.1016/j.jaci.2016.07.038 27639934

[85] Sears MR (2002). Adverse effects of beta-agonists. J Allergy Clin Immunol.

[86] Chan W-L, Yang K-P, Chao T-F, Huang C-C, Huang P-H, Chen Y-C, et al. The association of asthma and atrial fibrillation — A nationwide population-based nested case–control study. Int J Cardiol. 2014 2014/09/20/; 176(2):464–469. 10.1016/j.ijcard.2014.07.087 25127961

[87] Thottathil P, Acharya J, Moss AJ, Jons C, McNitt S, Goldenberg I, et al. Risk of Cardiac Events in Patients With Asthma and Long-QT Syndrome Treated With Beta2 Agonists. Am J Cardiol. 2008 2008/10/01/; 102(7):871–874. 10.1016/j.amjcard.2008.05.029 PMC4005827 18805113

[88] Appleton SL, Ruffin RE, Wilson DH, Taylor AW, Adams RJ. Cardiovascular disease risk associated with asthma and respiratory morbidity might be mediated by short-acting β2-agonists. J Allerg Clin Immunol. 2009 2009/01/01/; 123(1):124–130.e1. 10.1016/j.jaci.2008.10.032 19130933

[89] Adimadhyam S, Schumock GT, Walton S, Joo M, McKell J, Lee TA (2014). Risk of arrhythmias associated with ipratropium bromide in children, adolescents, and young adults with asthma: a nested case-control study. Pharmacotherapy..

[90] Calzetta L, Hanania NA, Dini FL, Goldstein MF, Fairweather WR, Howard WW, et al. Impact of doxofylline compared to theophylline in asthma: A pooled analysis of functional and clinical outcomes from two multicentre, double-blind, randomized studies (DOROTHEO 1 and DOROTHEO 2). Pulmon Pharmacol Ther. 2018 2018/12/01/; 53:20–26. 10.1016/j.pupt.2018.09.007 30219705

[91] Coleman JJ, Vollmer WM, Barker AF, Schultz GE, Buist AS. Cardiac Arrhythmias during the Combined Use of β-Adrenergic Agonist Drugs and Theophylline. Chest. 1986 1986/07/01/; 90(1):45–51. 10.1378/chest.90.1.45 2873000

[92] Mohammad HA, Abdulfttah MT, Abdulazez AO, Mahmoud AM, Emam RM. A study of electrolyte disturbances in patients with chronic stable asthma and with asthma attacks. Egypt J Chest Dis Tuberc. 2014 2014/07/01/;63(3):529–534.

[93] Crowell MD, Zayat EN, Lacy BE, Schettler-Duncan A, Liu MC. The Effects of an Inhaled β2-Adrenergic Agonist on Lower Esophageal Function: A Dose-Response Study. Chest. 2001 2001/10/01/; 120(4):1184–1189. 10.1378/chest.120.4.1184 11591558

[94] van Soest EM, Dieleman JP, Kuipers EJ (2008). The effect of anticholinergic agents on gastro-oesophageal reflux and related disorders. Expert Opin Drug Saf.

[95] Lee C-H, Choi S, Jang EJ, Kim D-W, Yoon HI, Kim YJ, et al. The effects of inhaled respiratory drugs on the risk of stroke: A nested case-control study. Pulmon Pharmacol Ther. 2016 2016/10/01/; 40:7–14. 10.1016/j.pupt.2016.07.002 27418383

[96] Zarar A, Khan AA, Adil MM, Qureshi AI. Anaphylactic shock associated with intravenous thrombolytics. Am J Emerg Med. 2014; 32(1): 113 e3–5. PubMed PMID: 24091200. Epub 2013/10/05. eng. 10.1016/j.ajem.2013.08.046 24091200

[97] King C, McKenna A, Farzan N, Vijverberg SJ, van der Schee MP, Maitland-van der Zee AH, et al. Pharmacogenomic associations of adverse drug reactions inasthma: systematic review and research prioritisation. Pharma J. 2020 2020/10/01; 20(5):621–628. 10.1038/s41397-019-0140-y PMC7502355 31949291

[98] Aagaard L, Hansen EH. Adverse drug reactions associated with asthma medications in children: systematic review of clinical trials. Int J Clin Pharm 2014;36(2):243–252. PubMed PMID: 24562976. Epub 2014/02/25. eng. 10.1007/s11096-014-9924-y 24562976

[99] Muruet W, Rudd A, Wolfe CDA, Douiri A. Long-Term Survival After Intravenous Thrombolysis for Ischemic Stroke: A Propensity Score-Matched Cohort With up to 10-Year Follow-Up. Stroke. 2018;49(3):607–613. PubMed PMID: 29440582. Epub 02/12. eng. 10.1161/STROKEAHA.117.019889 PMC5839705 29440582

[100] de Weerd L, Luijckx G-JR, Groenier KH, van der Meer K. Quality of life of elderly ischaemic stroke patients one year after thrombolytic therapy. A comparison between patients with and without thrombolytic therapy. BMC Neurol. 2012; 12:61. PubMed PMID: 22835054. eng. 10.1186/1471-2377-12-61 PMC3444943 22835054

[101] Randall KL, Hawkins CA. Antihistamines and allergy. Aust Prescr. 2018; 41(2):41–45. PubMed PMID: 29670310. Epub 04/03. eng. 10.18773/austprescr.2018.013 PMC5895478 29670310

[102] Brown SG. Clinical features and severity grading of anaphylaxis. J Allergy Clin Immunol 2004;114(2):371–376. PubMed PMID: 15316518. Epub 2004/08/19. eng. 10.1016/j.jaci.2004.04.029 15316518

[103] O’Carroll CB, Aguilar MI (2015). Management of Postthrombolysis Hemorrhagic and Orolingual Angioedema Complications. Neurohospitalist.

[104] Khatri P, Levine J, Jovin T. Intravenous Thrombolytic Therapy For Acute Ischemic Stroke. Continuum. 2008;14(6):46–60. PubMed PMID: 00132979–200812000-00005.

[105] Jauch EC, Saver JL, Adams HP, Jr., Bruno A, Connors JJ, Demaerschalk BM, et al. Guidelines for the early management of patients with acute ischemic stroke: a guideline for healthcare professionals from the American Heart Association/American Stroke Association. Stroke. 2013;44(3):870–947. PubMed PMID: 23370205. Epub 2013/02/02. eng. 10.1161/STR.0b013e318284056a 23370205

[106] Kim S-D, Cho K-S. Samter's Triad: State of the Art. Clin Exp Otorhinolaryngol. 2018; 11(2):71–80. PubMed PMID: 29642688. Epub 04/13. eng. 10.21053/ceo.2017.01606 PMC5951071 29642688

[107] Zuckerman SL, Seder DB, Tsujiura C, Cushing D, Gallup H, Mocco J, et al. Aspirin allergy desensitization in cerebrovascular disease. A report of two cases, literature review and management guide for the neurointerventionalist. Interv Neuroradiol. 2014; 20(1):5–11. PubMed PMID: 24556294. Epub 02/10. eng. 10.15274/INR-2014-10002 PMC3971141 24556294

[108] Ramanuja S, Breall JA, Kalaria VG (2004). Approach to “Aspirin Allergy” in Cardiovascular Patients. Circulation..

[109] Nam NJ, Chau D, Miller RL, Canfield SM (2006). Desensitization to Clopidogrel and Ticlopidine in two patients with coronary artery disease and indwelling drug-eluting stents. J Allergy Clin Immunol.

[110] Chupp GL (2008). Say what, Beta-blockers for asthma?. Am J Respir Cell Mol Biol.

[111] Brooks TW, Creekmore FM, Young DC, Asche CV, Oberg B, Samuelson WM. Rates of hospitalizations and emergency department visits in patients with asthma and chronic obstructive pulmonary disease taking beta-blockers. Pharmacotherapy. 2007;27(5):684–690. PubMed PMID: 17461703. Epub 2007/04/28. eng. 10.1592/phco.27.5.684 17461703

[112] van der Woude HJ, Zaagsma J, Postma DS, Winter TH, van Hulst M, Aalbers R. Detrimental effects of beta-blockers in COPD: a concern for nonselective beta-blockers. Chest. 2005;127(3):818–824. PubMed PMID: 15764762. Epub 2005/03/15. eng. 10.1378/chest.127.3.818 15764762

[113] Morales DR, Lipworth BJ, Donnan PT, Jackson C, Guthrie B. Respiratory effect of beta-blockers in people with asthma and cardiovascular disease: population-based nested case control study. BMC Med. 2017; 15(1):18-. PubMed PMID: 28126029. eng. 10.1186/s12916-017-0781-0 PMC5270217 28126029

[114] Kaufman J, Schmitt S, Barnard J, Busse W (1992). Angiotensin-converting enzyme inhibitors in patients with bronchial responsiveness and asthma. Chest..

[115] Christiansen SC, Zuraw BL (2019). Treatment of hypertension in patients with asthma. N Engl J Med.

[116] Chiu KY, Li JG, Lin Y. Calcium channel blockers for lung function improvement in asthma: A systematic review and meta-analysis. Ann Allerg Asthma Immunol. 2017; 119(6):518–523.e3. 10.1016/j.anai.2017.08.013 29032888

[117] Chiang KL, Kuo FC, Lee JY, Huang CY. Association of epilepsy and asthma: a population-based retrospective cohort study. PeerJ. 2018;6:e4792. PubMed PMID: 29796346. Pubmed Central PMCID: PMC5961633. Epub 2018/05/26. eng. 10.7717/peerj.4792 PMC5961633 29796346

[118] Lomia M, Chapichadze Z, Pruidze M, Platonov P. Efficacy Of Monotherapy With Carbamazepine And Valproic Acid In Patients With Bronchial Asthma: Is Asthma A Neurological Disease? Int J Neurol. 2004;4(1).

[119] Jain S, Jain KC. Effect of Phenytoin Sodium in the Management of Poorly Controlled Bronchial Asthma at a Rural Health Center in Phalodi, Rajasthan, India. J Asthma. 1991 1991/01/01; 28(3):201–211. 10.3109/02770909109082748 2071554

[120] Badawy MS, Ismail SM, Abass MA. Efficacy of Carbamazepine in treatment of bronchial asthma. Egypt J Chest Dis Tuberc. 2014 2014/01/01/;63(1):15–20.

[121] Slaton RM, Thomas RH, Mbathi JW (2013). Evidence for therapeutic uses of nebulized Lidocaine in the treatment of intractable cough and asthma. Ann Pharmacother.

[122] Tamagawa E, van Eeden SF. Impaired Lung Function and Risk for Stroke: Role of the Systemic Inflammation Response? Chest. 2006 2006/12/01/;130(6):1631–1633. 10.1378/chest.130.6.1631 17166971

[123] Rydell A, Janson C, Lisspers K, Ställberg B, Nowak C, Carlsson AC, et al. Endothelial dysfunction is associated with impaired lung function in two independent community cohorts. Respir Med. 2018 2018/10/01/;143:123–128. 10.1016/j.rmed.2018.09.009 30261983

[124] Gurgone D, McShane L, McSharry C, Guzik TJ, Maffia P. Cytokines at the Interplay Between Asthma and Atherosclerosis? Front Pharmacol. 2020;11:166. PubMed PMID: 32194407. eng. 10.3389/fphar.2020.00166 PMC7064545 32194407

[125] Ingebrigtsen TS, Marott JL, Vestbo J, Nordestgaard BG, Lange P. Coronary heart disease and heart failure in asthma, COPD and asthma-COPD overlap. BMJ Open Respir Res. 2020;7(1):e000470. PubMed PMID: PMC7011896. eng. 10.1136/bmjresp-2019-000470 PMC7011896 33371008

[126] Robba C, Bonatti G, Battaglini D, Rocco PRM, Pelosi P. Mechanical ventilation in patients with acute ischaemic stroke: from pathophysiology to clinical practice. Crit Care. 2019; 23(1):388-. PubMed PMID: 31791375. eng. 10.1186/s13054-019-2662-8 PMC6889568 31791375

[127] Ezeugwu VE, Olaogun M, Mbada CE, Adedoyin R (2013). Comparative lung function performance of stroke survivors and age-matched and sex-matched controls. Physiother Res Int.

[128] Jung KJ, Park JY, Hwang DW, Kim JH (2014). Ultrasonographic diaphragmatic motion analysis and its correlation with pulmonary function in hemiplegic stroke patients. Ann Rehabil Med.

[129] Austin V, Ku JM, Miller AA, Vlahos R. Ischaemic stroke in mice induces lung inflammation but not acute lung injury. Sci Rep. 2019;9(1):3622. PubMed PMID: 30842652. eng. 10.1038/s41598-019-40392-1 PMC6403328 30842652

[130] Samary CS, Ramos AB, Maia LA, Rocha NN, Santos CL, Magalhães RF, et al. Focal ischemic stroke leads to lung injury and reduces alveolar macrophage phagocytic capability in rats. Crit Care. 2018; 22(1): 249. PubMed PMID: 30290827. eng. 10.1186/s13054-018-2164-0 PMC6173845 30290827

[131] Zhao J, Xuan N-X, Cui W, Tian B-P. Neurogenic pulmonary edema following acute stroke: The progress and perspective. Biomed Pharmacother. 2020 2020/10/01/; 130:110478. 10.1016/j.biopha.2020.110478 32739737

[132] Courties G, Frodermann V, Honold L, Zheng Y, Herisson F, Schloss MJ, et al. Glucocorticoids Regulate Bone Marrow B Lymphopoiesis After Stroke. Circ Res. 2019;124(9):1372–85. PubMed PMID: 30782088. eng. 10.1161/CIRCRESAHA.118.314518 PMC6483874 30782088

[133] de Montmollin E, Ruckly S, Schwebel C, Philippart F, Adrie C, Mariotte E, et al. Pneumonia in acute ischemic stroke patients requiring invasive ventilation: Impact on short and long-term outcomes. J Infect. 2019 2019/09/01/; 79(3):220–227. 10.1016/j.jinf.2019.06.012 31238051

[134] Wu F, Liu Y, Ye G, Zhang Y. Respiratory Muscle Training Improves Strength and Decreases the Risk of Respiratory Complications in Stroke Survivors: A Systematic Review and Meta-analysis. Arch Phys Med Rehabil. 2020 2020/11/01/;101(11):1991–2001. 10.1016/j.apmr.2020.04.017 32445847

[135] Dhanesha N, Chorawala MR, Jain M, Bhalla A, Thedens D, Nayak M, et al. Fn-EDA (Fibronectin Containing Extra Domain A) in the Plasma, but Not Endothelial Cells, Exacerbates Stroke Outcome by Promoting Thrombo-Inflammation. Stroke. 2019;50(5):1201–1209. PubMed PMID: 30909835. eng. 10.1161/STROKEAHA.118.023697 PMC6476677 30909835

[136] Satou Y, Oguro H, Murakami Y, Onoda K, Mitaki S, Hamada C (2013). Gastroesophageal reflux during enteral feeding in stroke patients: a 24-hour esophageal pH-monitoring study. J Stroke Cerebrovasc Dis.

[137] Martino R, Foley N, Bhogal S, Diamant N, Speechley M, Teasell R (2005). Dysphagia after stroke. Stroke..

[138] Zechariah JA (2020). Prevalence and types of pulmonary disability in patients with Gastroesophageal reflux disease (GERD). Int J Dermatopathol Surg.

[139] Ondeto PAF, Moussavou IFM, Nzenze JR, Moussavou JB, Patrice Emery Itoudi Bignoumba DM (2020). Impact of typical gastroesophageal reflux in patients with asthma. Int J Inform Res Rev.

[140] Arai N, Nakamizo T, Ihara H, Koide T, Nakamura A, Tabuse M (2017). Histamine H2-blocker and proton pump inhibitor use and the risk of pneumonia in acute stroke: a retrospective analysis on susceptible patients. PLoS One.

[141] Hoffmann S, Harms H, Ulm L, Nabavi DG, Mackert BM, Schmehl I (2017). Stroke-induced immunodepression and dysphagia independently predict stroke-associated pneumonia - the PREDICT study. J Cereb Blood Flow Metab.

[142] Teh WH, Smith CJ, Barlas RS, Wood AD, Bettencourt-Silva JH, Clark AB (2018). Impact of stroke-associated pneumonia on mortality, length of hospitalization, and functional outcome. Acta Neurol Scand.

[143] Haeusler K, Herm J, Konieczny M, Grittner U, Lainscak M, Endres M (2015). Impact of chronic inflammatory airway disease on stroke severity and long-term survival after ischemic stroke - a retrospective analysis. BMC Neurol.

[144] Bianco A, Nigro E, Monaco ML, Matera MG, Scudiero O, Mazzarella G, et al. The burden of obesity in asthma and COPD: Role of adiponectin. Pulmon Pharmacol Ther. 2017 2017/04/01/;43:20–25. 10.1016/j.pupt.2017.01.004 28115224

[145] Scott HA, Gibson PG, Garg ML, Wood LG (2011). Airway inflammation is augmented by obesity and fatty acids in asthma. Eur Respir J.

[146] McGarry ME, Castellanos E, Thakur N, Oh SS, Eng C, Davis A, et al. Obesity and Bronchodilator Response in Black and Hispanic Children and Adolescents With Asthma. Chest. 2015 2015/06/01/; 147(6):1591–1598. 10.1378/chest.14-2689 PMC4451713 25742612

[147] Singh SS, Pilkerton CS, Shrader CD, Frisbee SJ. Subclinical atherosclerosis, cardiovascular health, and disease risk: is there a case for the Cardiovascular Health Index in the primary prevention population? BMC Public Health. 2018 2018/04/02; 18(1):429. 10.1186/s12889-018-5263-6 PMC5880087 29609588

[148] de Boer JD, Majoor CJ, van’t Veer C, Bel EH, van der Poll T (2012). Asthma and coagulation. Blood.

[149] Pitchford S, Cleary S, Arkless K, Amison R. Pharmacological strategies for targeting platelet activation in asthma. Curr Opin Pharmacol. 2019 2019/06/01/; 46:55–64. 10.1016/j.coph.2019.03.012 31026626

[150] Bradbury P, Traini D, Ammit AJ, Young PM, Ong HX. Repurposing of statins via inhalation to treat lung inflammatory conditions. Adv Drug Deliv Rev. 2018 2018/08/01/; 133:93–106. 10.1016/j.addr.2018.06.005 29890243

[151] Chou R, Dana T, Blazina I, Daeges M, Jeanne TL (2016). Statins for prevention of cardiovascular disease in adults: evidence report and systematic review for the US preventive services task force. JAMA..

[152] Maneechotesuwan K, Ekjiratrakul W, Kasetsinsombat K, Wongkajornsilp A, Barnes PJ (2010). Statins enhance the anti-inflammatory effects of inhaled corticosteroids in asthmatic patients through increased induction of indoleamine 2, 3-dioxygenase. J Allergy Clin Immunol.

[153] Zeki AA, Elbadawi-Sidhu M. Innovations in asthma therapy: is there a role for inhaled statins? Expert Rev Respir Med 2018;12(6):461–473. PubMed PMID: 29575963. Epub 2018/05/03. eng. 10.1080/17476348.2018.1457437 PMC6018057 29575963

[154] Zozina VI, Covantev S, Kukes VG, Corlateanu A. Coenzyme Q10 in COPD: an unexplored opportunity? COPD: J Chron Obstruct Pulmon Dis. 2021:1–17. 10.1080/15412555.2020.1849084 33441012

[155] Kim HA, Perrelli A, Ragni A, Retta F, De Silva TM, Sobey CG, et al. Vitamin D Deficiency and the Risk of Cerebrovascular Disease. Antioxidants (Basel). 2020; 9(4):327. PubMed PMID: 32316584. eng. 10.3390/antiox9040327 PMC7222411 32316584

[156] Sutherland ER, Goleva E, Jackson LP, Stevens AD, Leung DYM. Vitamin D levels, lung function, and steroid response in adult asthma. Am J Respir Crit Care Med 2010;181(7):699–704. PubMed PMID: 20075384. Epub 2010/01/14. eng. 10.1164/rccm.200911-1710OC PMC2868500 20075384

[157] Searing DA, Zhang Y, Murphy JR, Hauk PJ, Goleva E, Leung DY (2010). Decreased serum vitamin D levels in children with asthma are associated with increased corticosteroid use. J Allergy Clin Immunol.

[158] Wang Y, Ji H, Tong Y, Zhang Z-B. Prognostic Value of Serum 25-Hydroxyvitamin D in Patients with Stroke. Neurochem Res. 2014 2014/07/01; 39(7):1332–1337. 10.1007/s11064-014-1316-0 24789365

[159] Qiu H, Wang M, Mi D, Zhao J, Tu W, Liu Q. Vitamin D status and the risk of recurrent stroke and mortality in ischemic stroke patients: Data from a 24-month follow-up study in China. J Nutr Health Aging. 2017 2017/07/01; 21(7):766–771.10.1007/s12603-016-0821-z28717806

[160] Staats P, Emala C, Simon B, Errico JP. Chapter 111 - Neurostimulation for asthma. In: Krames ES, Peckham PH, Rezai AR, editors. Neuromodulation. 2nd ed: Academic Press; 2018. p. 1339–45.

